# Prevalence of vasculitis, systemic lupus erythematosus, rheumatoid arthritis, systemic sclerosis, idiopathic inflammatory myopathies and spondyloarthritis in Australia: a systematic review and meta‐analysis

**DOI:** 10.1111/imj.70210

**Published:** 2025-09-15

**Authors:** L. Roper, E. Cameron, T. Yu, N. Li, M. Parker, N. Nassar, M. Nikpour

**Affiliations:** ^1^ Sydney School of Public Health The University of Sydney Sydney New South Wales Australia; ^2^ Sydney Musculoskeletal Research Centre, Faculty of Medicine and Health The University of Sydney Sydney New South Wales Australia; ^3^ Leeder Centre for Health Policy, Economics and Data, Faculty of Medicine and Health The University of Sydney Sydney New South Wales Australia; ^4^ Department of Rheumatology Royal Prince Alfred Hospital Sydney New South Wales Australia; ^5^ Department of Rheumatology, Westmead, Blacktown and Auburn Hospital Western Sydney Local Health District Sydney New South Wales Australia; ^6^ Institute of Academic Medicine Royal Prince Alfred Hospital Sydney New South Wales Australia; ^7^ Children's Hospital at Westmead Clinical School, Faculty of Medicine and Health The University of Sydney Sydney New South Wales Australia; ^8^ Charles Perkins Centre The University of Sydney Sydney New South Wales Australia

**Keywords:** systemic autoimmune rheumatic diseases, prevalence, systematic review, meta‐analysis, Australia, administrative health data

## Abstract

**Background:**

Systemic autoimmune rheumatic diseases (SARDs), including vasculitis, systemic lupus erythematosus (SLE), rheumatoid arthritis (RA), systemic sclerosis (SSc), idiopathic inflammatory myopathies (IIM), spondyloarthritis (SpA), Sjogrens disease (SjD) and mixed connective tissue disease (MCTD), are rare but associated with substantial morbidity and healthcare burden. Their clinical heterogeneity and diagnostic complexity pose challenges for epidemiological study.

**Objective:**

To summarise Australian data on the incidence or prevalence of the SARDs listed above.

**Methods:**

Scientific databases were searched in July 2024. Studies reporting SARD incidence or prevalence in the general Australian population were included. Where three or more studies of the same disease existed within a comparable population, which were not at high risk of bias (Hoy Tool), a random effects model was fitted.

**Results:**

From 1357 publications, 58 were included, on: vasculitis (16), SLE (14), RA (10), SSc (nine), IIM (six) and SpA (three), with none on SjD or MCTD identified. Meta‐analysis was possible in ANCA‐associated vasculitis (AAV) (incidence 10.80 per million person‐years), SLE (prevalence 57.86 per 100 000), lupus nephritis (prevalence 11.20 per 100 000, incidence 1.02 per 100 000 person‐years), SSc (prevalence 25.58 per 100 000), IIM (incidence 9.96 per million person‐years) and inclusion body myositis (prevalence 27.73 per million). Data were limited for paediatric populations and specific at‐risk groups (e.g. Australians of Asian heritage).

**Conclusion:**

Australia may have above‐average prevalence of SLE and SSc and geographic variation in SSc and AAV. Key research gaps include: (1) sparse data for non‐AAV vasculitis, RA, SpA, SjD, MCTD and children; (2) limited validated algorithms to identify SARDs in administrative health data; and (3) a need for large‐scale spatial studies to detect disease clusters.

## Introduction

Systemic autoimmune rheumatic diseases (SARDs) can result in prolonged morbidity and substantial healthcare utilisation.^1^, ^2^, ^3^ Current data suggest that Australia has an above‐average prevalence of several SARDs^4^, ^5^, ^6^, ^7^, ^8^, ^9^, ^10^, ^11^ and possible geographic variation in prevalence, perhaps driven by environmental exposures^4^, ^9^, ^12^, ^13^, ^14^, ^15^, ^16^, ^17^, ^18^, ^19^, ^20^ Accurate national SARD prevalence estimates can inform healthcare system planning and establish reference points for identifying geographic or temporal clusters.

This systematic literature review and meta‐analysis summarises available data on the Australian incidence or prevalence of multiple SARDs, specifically vasculitis (including ANCA‐associated vasculitis (AAV), Takayasu arteritis, giant cell arteritis (GCA), IgA vasculitis and Behcet disease), systemic lupus erythematosus (SLE), rheumatoid arthritis (RA), systemic sclerosis (SSc), idiopathic inflammatory myopathies (IIM), spondyloarthritis (SpA), Sjogren disease (SjD) and mixed connective tissue disease (MCTD). Given their rarity and the challenges in diagnosis (evolving criteria, heterogenous disease manifestations), SARD prevalence estimates are difficult to obtain, and shared insights can be drawn from examining the methodologies used.

Our objectives were to: (1) synthesise Australian studies of incidence or prevalence of the listed SARD, (2) generate pooled incidence or prevalence estimates where possible and (3) identify opportunities for future research.

## Methods

### Study design

This study followed the Joanna Briggs Institute guidance for prevalence reviews,^21^ and reporting follows the Preferred Reporting Items for Systematic Reviews and Meta‐Analyses (PRISMA)^22^ checklist.

### Eligibility criteria

We included peer‐reviewed studies and grey literature published prior to July 2024 that: (1) involved Australian children or adults; (2) identified SARDs by self‐report, clinician diagnosis or administrative data; and (3) reported or provided sufficient data to calculate SARD incidence or prevalence.

We excluded studies that: (1) reported SARD frequency only within specific conditions (i.e. pregnancy)^23^, ^24^, ^25^ and (2) were duplicate publications of identical data, including conference abstracts with a subsequent paper, literature reviews and secondary analyses of Australian Bureau of Statistics (ABS) National Health Surveys or Global Burden of Disease data.^2^, ^3^, ^26^, ^27^ Several studies had overlapping but non‐identical populations (e.g. same site, different years or case definitions). To avoid regional over‐representation, we performed sensitivity analyses comparing inclusion of one or both studies. Both were retained only if they shifted the pooled estimate in opposite directions or had minimal impact in the same direction (defined as <10% relative change, <2 per 100 000 absolute change and within the original 95% confidence interval (CI)). When only one was included, the more recent study was preferred. Where studies covered overlapping but highly distinct catchments (e.g. state‐wide vs single hospital^28^, ^29^), both were included.

### Information sources

Medline, EMBASE, CINAHL, Web of Science, Scopus, Google Scholar and Google (to identify grey literature) databases were searched on 1 July 2024. The search strategy (Section S1 (S1)) was developed with an academic librarian.

### Selection process and data collection

The first author (LR) screened all abstracts to exclude clearly non‐relevant articles, with the remainder moving to full text review. The full texts were independently screened by two authors (LR and EC or TY), with discrepancies resolved with the senior author (MN). Data from included studies were extracted into a template using Covidence software.^30^

### Risk‐of‐bias assessment

We assessed study quality using the Hoy Risk of Bias Tool, which is appropriate for prevalence studies^31^ and provides a summary score (0–9) and overall ratings of low, moderate or high risk of bias.

### Statistical analysis

Studies were synthesised narratively, and key results are summarised in Sections S2–S7.

A meta‐analysis was performed when three or more studies reported on the same disease in comparable populations and were not high risk of bias. To account for potential differences in disease patterns, meta‐analyses were conducted separately for paediatric and adult populations. When reported denominators were descriptive (i.e. ‘population of South Australia’), population data from the ABS was used for model weighting. For studies reporting incidence rates without CIs or population data, these values were estimated assuming a Poisson distribution.

Incidence and prevalence were standardised to cases per 100 000 person‐years and per 100 000 population, unless otherwise specified (vasculitis and IIM are reported per million due to rarity, and inflammatory arthritis is reported in percent due to high prevalence). Where disease definitions varied, results were disaggregated into comparable subgroups (e.g. IIM subtypes).

To account for suspected regional differences and low prevalence data, a random‐effects model (with inverse variance weighting and Freeman‐Tukey double arcsine transformation) was applied to prevalence data. For diseases with incidence data, a random‐effects model was fitted to log‐transformed incidence data. If significant heterogeneity was present (identified by a wide prediction interval), a subgroup analysis was performed to investigate the cause. This approach has been recommended over the use of Tau2 or I^2^ in prevalence or incidence meta‐analysis.^32^, ^33^

Studies reporting point or 5‐year period prevalence were included, as SARDs are chronic with stable incidence and mortality; both measures reflect the same underlying frequency. Sensitivity analyses showed that 5‐year estimates did not introduce heterogeneity. The influence of outlying results was assessed by sequentially excluding these studies in sensitivity analyses. The GRADE assessment^34^ was not applied as these were observational data. Certainty of evidence was assessed by considering risk of bias and heterogeneity.^34^ High‐risk studies were excluded from the meta‐analysis to improve confidence in the results. Statistical analyses were performed in R, version 4.4.0 (R Foundation for Statistical Computing), using the meta package.

## Results

### Study selection

**Figure 1:**
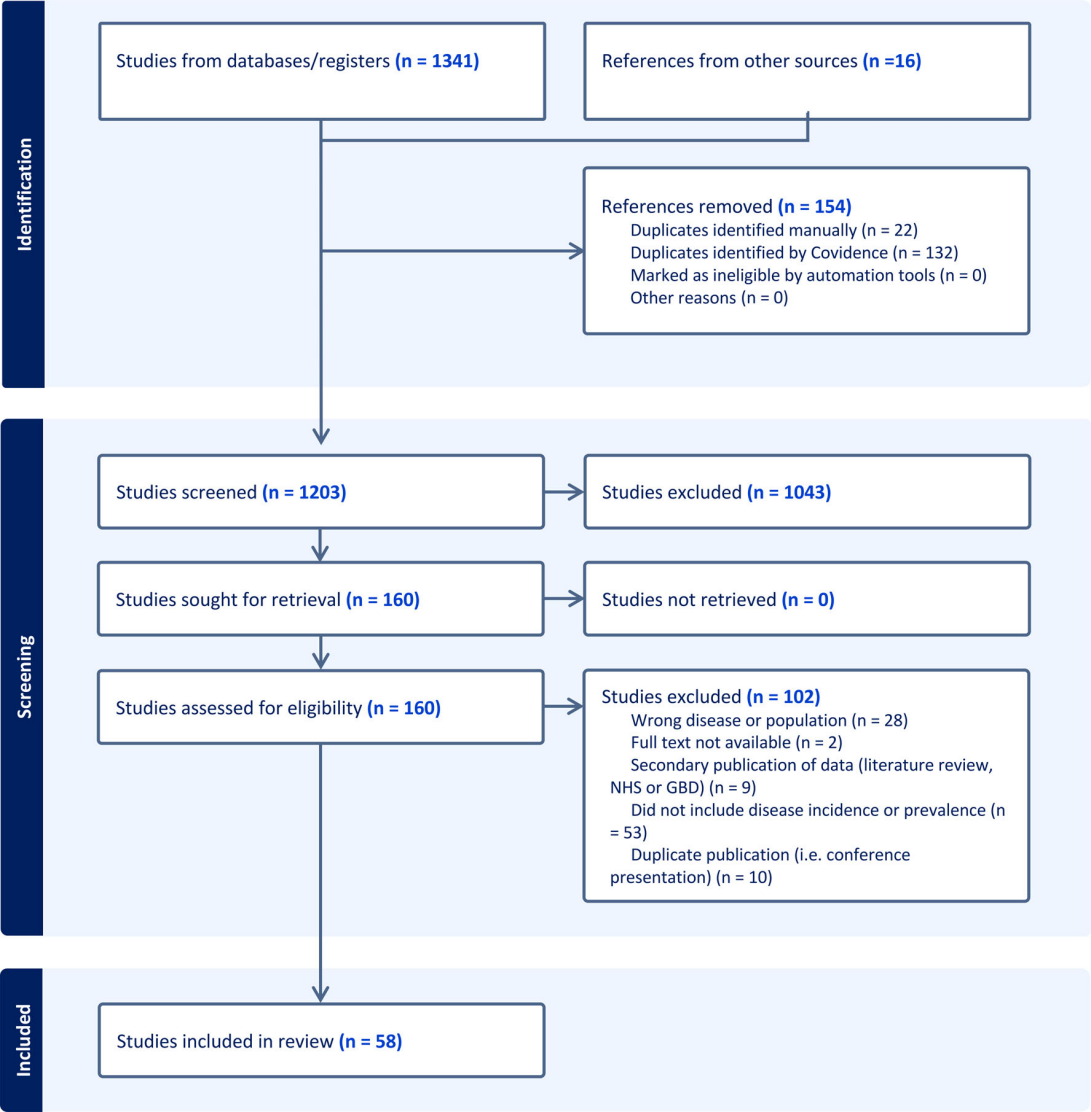
The study selection process. GBD, Global Burden of Disease; NHS, National Health Service.

Figure 1 demonstrates the selection process. From an initial pool of 1357 studies, 1197 were excluded at abstract screening as they clearly did not meet inclusion criteria or were duplicates. A further 102 were excluded after full text review, for the reasons outlined in Figure 1, leaving 58 studies.

**Figure 2:**
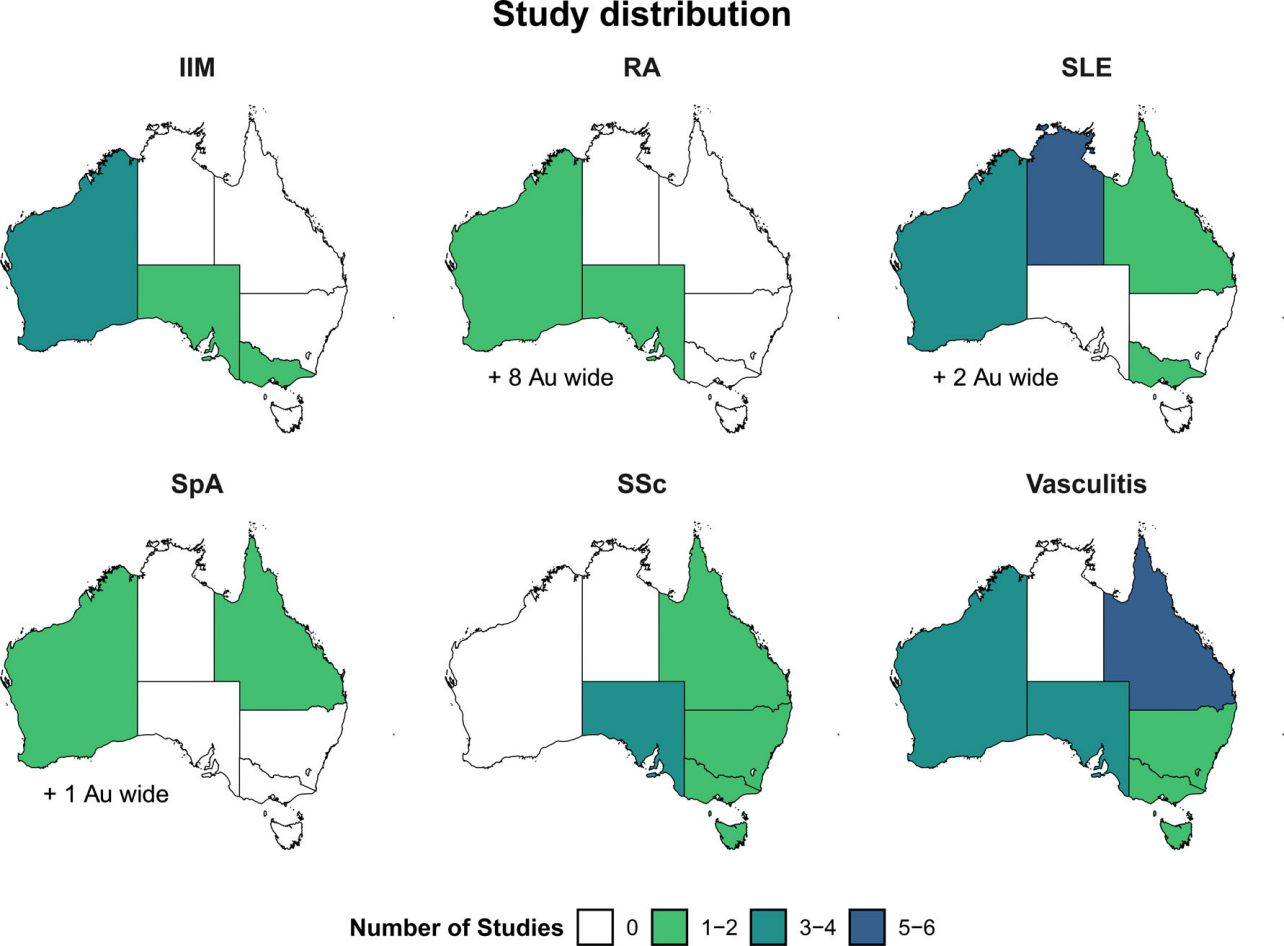
Distribution of incidence and prevalence studies across Australia, organised by disease type. IIM, inflammatory myopathies; RA, rheumatoid arthritis; SLE, systemic lupus erythematosus; SpA, spondyloarthritis; SSc, systemic sclerosis.

Figure 2 illustrates the SARD incidence and prevalence data by State and Territory, and Table 1 summarises the number of included studies by disease and how many were suitable for meta‐analysis.

**Table 1:** Total studies identified

Disease	Number of studies	Number of studies included in the meta‐analysis	Pooled (meta‐analysed) or reported (non‐meta‐analysed) disease frequency
Vasculitis	16	7 (AAV incidence)	10.80 per million person‐years
SLE	14 (7 LN only)	SLE: 4, LN prevalence: 3, LN incidence: 4*	SLE: 57.86 per 100 000 LN prevalence: 11.20 per 100 000 LN incidence: 1.02 per 100 000 person‐years
RA	10	0	0.34–3.9 per 100
SSc	9	9	25.58 per 100 000
IIM	6	IIM: 3, DM, PM: 4, IBM: 4*	IIM: 9.96 per million person‐years (adjusted to: 7.86 per million person‐years after sensitivity analysis) IBM prevalence: 27.73 per million
SpA	3	0	NA: 3 separate diseases

* One study was excluded from the pooled estimate due to an overlapping population with another included study (see Supporting Information for details). AAV, ANCA‐associated vasculitis; DM, dermatomyositis; IBM, inclusion body myositis; IIM, inflammatory myopathies; LN, lupus nephritis; PM, polymyositis; RA, rheumatoid arthritis; SLE, systemic lupus erythematosus; SpA, spondyloarthritis; SSc, systemic sclerosis.

### Vasculitis

Sixteen studies on vasculitis were identified (Section S2.1), 12 of which focused on AAV, two on GCA, two on IgA vasculitis (one of which included a subgroup analysis of individuals younger than 20 years) and one on Takayasu arteritis. The prevalence of GCA was 32–54 per million (Section S2.2), and of Takayasu arteritis, 3.9 per million. The incidence of IgA vasculitis was 4.5–18 per million person‐years in adults and 25.9 in children (younger than 20 years) in the single study that included a relevant subgroup analysis.^35^ Of the 12 AAV studies, three reported prevalence only and three were excluded for high risk of bias, leaving seven studies for inclusion in the pooled incidence estimate.^36^, ^37^ For case identification, half of the AAV studies used renal biopsy registries; the remainder used hospital ICD codes. Challenges with biopsy‐based ascertainment are discussed later. Five studies reported variation in incidence, by geography (urban vs rural)^36^, ^37^ or before and after environmental events,^38^, ^39^, ^40^ suggesting possible links to environmental exposure. AAVs were analysed as a single group, due to heterogenous disease groupings, including ‘overall AAV’, ‘GPA, MPA, EGPA’^41^, ^42^ or ‘MPO‐ANCA, PR3‐ANCA’.^38^, ^39^, ^43^

#### Meta‐analysis

AAV incidence was estimated at 10.8 per million person‐years (95% CI: 7.79–14.96) (Section S2.3), based on the results of seven Australian studies in which the incidence ranged from 4.6 to 25.9 per million person‐years.^36^, ^37^, ^41^, ^42^, ^43^, ^44^, ^45^ This is slightly lower than the global pooled incidence of 17.2 per million person‐years (95% CI: 13.3–21.6).^46^

### SLE

Fourteen studies on SLE were identified, including seven on lupus nephritis (LN) only (Section S3.1). Of the seven SLE studies, two were paediatric and one had a high risk of bias,^13^, ^15^, ^47^ leaving insufficient data to meta‐analyse paediatric SLE prevalence and four studies to meta‐analyse adult SLE prevalence. All four studies were conducted in Far North Queensland, Central Australia, or the Top End of the Northern Territory,^7^, ^8^, ^9^, ^10^ limiting national generalisability due to distinct regional demographics—particularly the high proportion of Aboriginal and Torres Strait Islander peoples (10%–33% vs 3.8% nationally),^48^ who experience a high prevalence of SLE.^49^ Although all four studies reported subgroup analyses by Indigenous status, none examined other at‐risk groups, such as Australians of Asian heritage.

Six of the seven LN studies used renal biopsy databases for case identification; the seventh used ICD‐coded hospital data.^28^ Three reported subgroup analyses for Aboriginal and Torres Strait Islander peoples, and two reported a separate estimate for Australians of Asian heritage.^29^, ^50^

#### Meta‐analysis

The overall prevalence of SLE was 57.86 per 100 000 (95% CI: 33.12–89.19, prediction interval: 1.46–186.29) (Section S3.3), derived from four studies conducted in Northern Australia (NA), reporting a prevalence of 19 to 166 per 100 000.^7^, ^8^, ^9^, ^10^ Subgroup analysis revealed a prevalence of 29.95 per 100 000 (95% CI: 18.91–43.42) in non‐Indigenous Australians and 91.24 per 100 000 (95% CI: 52.72–139.92) in Aboriginal and Torres Strait Islander people. The two paediatric studies (not meta‐analysed) reported SLE annual incidence rates of 0.33–0.44 per 100 000 person‐years.^13^, ^47^

The overall prevalence of LN was 11.2 per 100 000 (95% CI: 5.1–19.7), derived from three studies, reporting a prevalence from 5.5 to 15 per 100 000^28^, ^29^, ^50^ (Section S3.4). Prevalence of LN in Australians of Asian heritage (not meta‐analysed due to insufficient data) was reported as 27.9–65.3 per 100 000.^29^, ^50^ The overall incidence of LN was 1.02 per 100 000 person‐years (95% CI: 0.50–2.08), derived from four studies^19^, ^28^, ^29^, ^51^ (the fifth was excluded due to population overlap^52^) (Section S3.5). Subgroup analysis revealed an overall incidence of 5.11 (95% CI: 3.36–7.77) in Aboriginal and Torres Strait Islander peoples and 0.47 (95% CI: 0.35–0.62) in non‐Indigenous people.

### Rheumatoid arthritis

Ten publications have examined RA prevalence in Australia (Section S4.1); however, a meta‐analysis was not possible due to heterogeneity and study quality (Section S4.2). Five editions of the National Health Survey (NHS) estimated RA prevalence at 2%–2.8% from 2009 to 2023^53^, ^54^, ^55^ but relied on self‐report, which overestimates RA prevalence, leading to a high risk‐of‐bias rating.^11^ A 1999 South Australia (SA) survey identified an RA prevalence of 0.9%, based on self‐report of medically confirmed cases.^56^

There are four other studies of RA prevalence. First, a linked data analysis of the Australian Longitudinal Study of Women's Health (ALSWH) and administrative health data^11^ compared five case identification techniques, reporting a prevalence of 1.1%–5.4%, confirming that self‐report over‐estimates prevalence. Although rigorous, the study was not incorporated into the meta‐analysis as it only included women.

The second study, conducted in Western Australia (WA), using hospital data and aggregated Pharmaceutical Benefits Scheme (PBS) data, estimated RA prevalence at 0.34%–0.72% for 1995–2014.^57^ These estimates were lower than those from the ALSWH study, likely due to stricter medication definitions and inclusion of males. The authors assumed that 50% of RA patients use biologic disease‐modifying antirheumatic drugs (bDMARDs), which may not account for increasing bDMARD use.^58^ Annual dispensing data were used as a proxy for unique cases, which may have resulted in double‐counting of individuals who used a bDMARD over multiple years.

The third, using the Medicine Insight Database, analysed de‐identified data from the electronic medical records of GP practices and calculated an RA prevalence of 0.9%; however, it included polymyalgia rheumatica in the RA definition.^59^ Finally, the oldest national data on Australian RA prevalence derive from the 1978–1982 National Health and Medical Research Council (NHMRC) twin registry, which had a moderate risk of bias due to non‐response rates, identifying 186 RA cases, with a prevalence of 0.4%.^60^

### Systemic sclerosis

Nine studies of SSc prevalence in Australia were identified (Section S5.1), reporting prevalence from 8.6 to 61.0 per 100 000.^4^, ^5^, ^6^, ^61^, ^62^, ^63^, ^64^, ^65^, ^66^ These nine studies represent five regions, due to overlapping populations from Cairns,^4^, ^66^ Tasmania^64^, ^65^ and SA.^5^, ^62^, ^63^

#### Meta‐analysis

Overall SSc prevalence was 25.58 per 100 000 (95% CI: 18–34) (Sections S5.2 and S5.3), slightly higher but with overlapping prediction intervals of the global prevalence of SSc (18.87 (1.5–25.28) per 100 000 persons^67^). Sensitivity analysis (Section S5.3) revealed minimal impact from inclusion of geographically overlapping populations. Two studies made comment on SSc cases being potentially increased in their region (Cairns and Edenhope, VIC),^4^, ^6^ yet a specific spatiotemporal analysis found no evidence for geographic clustering in SA.^63^

### Idiopathic inflammatory myopathies

The six studies of IIM used different disease definitions (Section S6.1). Synthesising the results of studies that use different definitions is challenging.^68^, ^69^ We approached this by disaggregating into IIM, dermatomyositis (DM), polymyositis (PM) and inclusion body myositis (IBM).

#### Meta‐analysis

The overall incidence of IIM was 9.96 (5.78–17.16) per million person‐years, based on three studies reporting rates of 7.4–19 per million person‐years,^12^, ^70^, ^71^ identifying cases from biopsy data alone,^12^ biopsy plus hospital data^70^ or ICD codes alone.^71^ As ICD codes may overestimate IIM,^68^ we assessed the impact of this study: excluding it reduced the overall incidence to 7.7 (7.1–8.3) per million person‐years (Section S6.3). The forest plot and individual incidence of PM and DM are in the Supporting Information (Sections S6.4 and S6.5).

The pooled prevalence of IBM was 27.73 (12.32–49.22) per million (Section S6.6), derived from three studies^12^, ^71^, ^72^ (the fourth was excluded due to population overlap^73^), identifying cases from biopsy data alone,^12^ biopsy data plus manual case finding from local clinicians^72^ or ICD codes alone.^71^

Although biopsy‐based studies have limitations (discussed later), the highest IBM prevalence was reported in a biopsy‐only study,^12^ whose authors attributed this high prevlaence to increasing diagnostic awareness, compared to historical studies.^12^ In contrast, however, the most recent study^71^ relied solely on ICD codes and reported a lower prevalence, possibly due to under‐ascertainment. The ICD‐10‐AM code used by this study to identify IBM cases from 2000 to 2017 (G72.4 ‘Inflammatory myopathy, not elsewhere classified’) is unvalidated in Australia and showed 0% sensitivity for IBM in a Norwegian study.^74^

### Spondyloarthritis

There were three studies of SpA: one of ankylosing spondylitis (AS), one of psoriatic arthritis (PsA) and one of ‘spondyloarthropathy’ (the definition of which included spondyloarthropathy, spondylosis and spondyloarthritis) (Sections S7.1 and S7.2). Due to the heterogeneity, meta‐analysis was not possible.

The prevalence of AS in WA was estimated to be 0.29%, based on aggregated PBS data for dispensing of tumour necrosis factor inhibitors with an authority code for AS.^75^ Data for PsA prevalence comes from one Aboriginal community in Queensland, which was extensively sampled using COPCORD methodology,^76^ identifying four cases of clinician‐confirmed PsA in a sample of 847, a point prevalence of 0.5%.^76^ Finally, data on the prevalence of spondyloarthropathy comes from a study of more than 1.5 million patients that was part of the Medicine Insight Database (de‐identified data extracted from the electronic medical records of more than 500 general physician practices^59^). They used broad inclusion criteria of any diagnosis of ‘spondylosis, spondylarthritis or spondyloarthropathy,’ which may have included age‐related degenerative disc disease. Their prevalence estimate was 1.5%.

## Discussion

This is the first systematic review and meta‐analysis of vasculitis, SLE, RA, SSc, IIM, SpA, SjD and MCTD incidence or prevalence in Australia, highlighting the challenges of generating high‐quality data for rare diseases with nuanced diagnostic criteria. Three research gaps were identified. First, prevalence/incidence data are limited for non‐ANCA‐associated vasculitis, SpA, RA, SLE in SA, MCTD and SjD. In those SARDs with existing prevalence/incidence estimates, there remains a paucity of data from some population groups, with limited studies in the paediatric population and Australians of Asian descent. Second, validation studies could strengthen the use of Australian administrative health data for SARD research. Finally, geographic variation in AAV, SLE and SSc prevalence may be considerable but requires further investigation.

Data on non‐ANCA‐associated vasculitis was limited, precluding meta‐analysis. Despite their relative frequency globally,^77^, ^78^ Australian data on SpA and PsA data are scarce. One WA study examined AS,^75^ one large primary care‐based study examined SpA but was limited by a fairly broad disease definition^59^ and PsA has been examined in a single, small Aboriginal community.^76^ SpA may be suited for analysis using Pharmaceutical Benefits Scheme (PBS) data.^79^ Whilst 10 studies reported RA prevalence, most had data limitations. Like SpA, RA is well‐suited to administrative data due to its characteristic medications, with a PBS algorithm proposed and partially validated.^11^ Most SLE studies were from NA, limiting national generalisability. We estimated SLE prevalence in NA to be 57.86 (33.12–89.19) per 100 000, higher than a recent global estimate of 43.7 per 100 000,^80^ but lower than contemporary US (65–81 per 100 000^81^) or UK (97–107 per 100 000^82^, ^83^) estimates. This may reflect under‐ascertainment, as most studies were over a decade old, relying on a single hospital or clinician sources, supplemented with manual case‐finding (see Supporting Information Section S3.1). Despite these limitations, existing data show that First Nations peoples are disproportionately affected, with SLE prevalence reaching 91.24 (52.72–139.92) per 100 000, reinforcing the need to reduce autoimmune disease severity experienced by First Nations Australians.^49^, ^84^, ^85^ To refine national SLE prevalence estimates, research must use more rigorous case‐finding, expand beyond the Northern Territory and Far North Queensland and examine other at‐risk groups including Australians of Asian descent.^29^, ^50^ The two studies examining Asian Australians reported an LN prevalence of 27.9 and 64.3 per 100 000, far exceeding the Australian estimate of 11.2 per 100 000.^29^, ^50^

Administrative health data support comprehensive case identification through population‐wide coverage and were used in multiple studies.^18^, ^28^, ^36^, ^71^, ^75^, ^86^, ^87^, ^88^ Limitations include inaccuracies and limitations in coverage, so administrative data are ideally validated against confirmed cases and non‐cases. Internationally, validation studies have assessed algorithms for RA,^89^ SLE,^90^ SpA,^91^, ^92^, ^93^ vasculitis^94^ and IIM^95^ identification, but administrative databases vary by jurisdiction; thus, such validation studies are not directly applicable to Australia. Only one Australian study validating administrative data for SARD research was identified, which reviewed ICD coding for RA in WA.^96^

Concordance between administrative and biopsy‐based studies of LN suggests administrative data may be a valid tool for LN identification. Six of seven studies used renal biopsy databases; one used ICD‐coded hospital admissions,^28^ with similar results to a biopsy‐based study from the same region.^97^

Biopsy registries were also commonly used for case ascertainment in AAV and IIM studies. Half of the AAV studies used renal biopsy databases. Although histopathology (e.g. lung, renal or ear/nose/throat biopsies) is the diagnostic gold standard,^98^ a Dutch study found that only 45% of AAV patients underwent renal biopsies,^99^ despite glomerulonephritis being reported in 65%.^98^, ^100^ The renal biopsy rate among the Australian AAV population is unknown, which could lead to an underestimation of incidence. The recent launch of the Australian and New Zealand Vasculitis Quality and Disease Registry (ANZVASC‐QDR) will strengthen future research.

As muscle biopsies are often reported at only a few laboratories, some studies screened all muscle biopsies in a given state for IIM case‐finding.^12^, ^70^, ^72^, ^101^ However, the 2017 European Alliance of Associations for Rheumatology/American College of Rheumatology (EULAR/ACR) criteria do not require muscle biopsy, particularly for adults with typical skin manifestations.^102^ Furthermore, magnetic resonance imaging (MRI) was not included in the EULAR/ACR classification criteria; however, there has been increasing interest in the role of MRI as a diagnostic tool to guide biopsy or, when combined with other clinical features, to be used in place of biopsy.^103^ The addition of MRI criteria to the EULAR/ACR criteria applied to a cohort of Australian patients resulted in improved diagnostic accuracy, even when biopsy was not used, compared to the original EULAR/ACR criteria.^104^ The proportion of patients currently diagnosed with IIM in Australia without a biopsy is unknown, however. A 1999 Victorian study found that 8.5% of IIM patients had no biopsy,^70^ and a 2007 SA study identified that 32% of DM (not IIM) patients had no biopsy,^101^ and including non‐biopsied hospitalised patients doubled the DM incidence estimates, from 1.4 to 2.9 per million person‐years.^101^ Improved availability and awareness of the role of MRI and extended antibody panels may mean biopsies are even less frequent now; thus, relying on biopsy data alone may underestimate prevalence. This may differ by subtype, as the importance of histological confirmation varies; for example, IBM lacks reliably associated autoantibodies and often requires biopsy for diagnosis. Some studies supplemented biopsy data by also sending letters to all relevant clinicians.^72^, ^73^ Given the heterogeneity of IIM and involvement of multiple specialties, these methods are logistically challenging.^72^, ^73^ As an alternative, administrative data offer broader coverage but come with challenges. IIM epidemiology is particularly difficult due to evolving classification systems, and ICD‐based case identification without clinical review can lead to overestimation^68^ in the case of DM and PM. In the case of IBM, ICD coding likely results in underestimation as there is no specific ICD‐10 code for IBM. These challenges underscore the value of establishing a national Australian IIM registry to facilitate research efforts^105^ and the potential role for MRI data as a source of case identification.

Geographic variation in disease frequency was reported in some studies of AAV,^36^, ^37^ SLE,^15^ IIM^70^ and SSc,^6^, ^66^ while others identified no geographic clustering.^41^, ^63^ This inconsistency may reflect weak geographic effects or selection bias. Random variation can cause some regions to appear to have higher or lower prevalence by chance, and clinician‐led studies may be biased toward areas with an observed excess of cases. When combined with small sample sizes, this effect can mimic true geographic differences. If SARD clusters do exist in Australia, they may be driven by local environmental exposures or regional demographic differences. Proposed exposures include silica dust (mining, quarrying, agriculture, forestry and construction), organic solvents and other chemicals.^106^, ^107^, ^108^, ^109^, ^110^, ^111^ National research using spatial statistics^112^, ^113^ may help to clarify these patterns.

### Strengths and limitations

The strengths of this paper are that it is based on a systematic literature search, informed by the JBI guidance^21^ and Preferred Reporting Items for Systematic Reviews and Meta‐Analyses (PRISMA) statement,^22^ and used a validated tool for appraising prevalence study bias.^31^ Limitations relate to the underlying data, particularly the number of regionally based studies, which may limit generalisability. While inclusion of overlapping studies mostly had minimal impact on pooled estimates, in some cases the effect was material, necessitating their exclusion. This further reduced the number of studies available for meta‐analysis and underscores the uneven geographic distribution of research across Australia, with some regions studied multiple times and others not at all. Additionally, certain population groups remain under‐represented, including paediatric populations and Australians of different ethnic backgrounds.

Most pooled estimates broadly aligned with international data, except SSc, where our estimate (25.58 per 100 000) exceeded the estimate for Australia (6.69 per 100 000) reported in a recent global meta‐analysis.^67^ The international estimate was based on three studies^4^, ^5^, ^61^ as opposed to our nine, as conference abstracts,^64^, ^66^, ^114^ registry studies^62^, ^63^ and regional studies^6^ were excluded. We are therefore confident in the robustness of our SSc estimate despite the difference from the global meta‐analysis.

## Conclusion

This paper provides pooled incidence or prevalence estimates for AAV, SLE, LN, SSc, IIM and IBM, contributing to a growing body of SARD research in Australia. We identified three opportunities for future research. First, there are limited data on the prevalence of non‐ANCA‐associated vasculitis, RA, SpA, SjD and MCTD in Australia and a general paucity of studies on SARD prevalence in the paediatric population and Australians of diverse ethnic backgrounds. Second, there are few validation studies of Australian administrative health data for SARD research. Third, given the challenge of accurately identifying true geographic clusters of a rare disease, larger studies using spatial statistics are needed to identify clusters, in turn yielding aetiological clues.

## Disclaimers

During the preparation of this work, generative AI was not used.

## Supporting information


**Data S1:** Supporting Information.

## References

[1] Gill TK , Mittinty MM , March LM , Steinmetz JD , Culbreth GT , Cross M . Global, regional, and national burden of other musculoskeletal disorders, 1990–2020, and projections to 2050: a systematic analysis of the Global Burden of Disease Study 2021. Lancet Rheumatol 2023; 5: e670–e682. 37927903 10.1016/S2665-9913(23)00232-1 PMC10620749

[2] Ackerman I , Gorelik A , Berkovic D , Buchbinder R . The Future Burden of Arthritis in Australia: Projections to the year 2040. 2024. 10.1016/S2665-9913(24)00247-9 39647490

[3] Ackerman IN , Pratt C , Gorelik A , Liew D . Projected burden of osteoarthritis and rheumatoid arthritis in Australia: a population‐level analysis. Arthritis Care Res 2018; 70: 877–883. 10.1002/acr.23414 28898565

[4] Abbot S , McWilliams L , Spargo L , de Costa C , Ur‐Rehman Z , Proudman S *et al*. Scleroderma in Cairns: an epidemiological study. Intern Med J 2020; 50: 445–452. 31157951 10.1111/imj.14376

[5] Chandran G , Smith M , Ahern MJ , Roberts‐Thomson PJ . A study of scleroderma in South Australia: prevalence, subset characteristics and nailfold capillaroscopy. Aust NZ J Med 1995; 25: 688–694. 10.1111/j.1445-5994.1995.tb02854.x 8770332

[6] Englert H , Joyner J , Bade R , Thompson M , Morris D , Chambers P *et al*. Systemic scleroderma: a spatiotemporal clustering. Intern Med J 2005; 35: 228–233. 15836501 10.1111/j.1445-5994.2005.00783.x

[7] Anstey NM , Bastian I , Dunckley H , Currie BJ . Systemic lupus erythematosus in Australian Aborigines: high prevalence, morbidity and mortality. Aust NZ J Med 1993; 23: 646–651. 10.1111/j.1445-5994.1993.tb04720.x 8141691

[8] Segasothy M , Phillips PA . Systemic lupus erythematosus in Aborigines and Caucasians in central Australia: a comparative study. Lupus 2001; 10: 439–444. 11434580 10.1191/096120301678646191

[9] Bossingham D . Systemic lupus erythematosus in the far north of Queensland. Lupus 2003; 12: 327–331. 12729060 10.1191/0961203303lu381xx

[10] Subramani P , Brady S , Thomas S , Pawar B . A retrospective analysis on systemic lupus erythematosus in the indigenous and nonindigenous population in central Australia focussing on treatment and outcomes of lupus nephritis. Lupus Sci Med 2017; 4: A92.

[11] Koller‐Smith L , Mehdi A , March L , Tooth L , Mishra GD , Thomas R . A novel method to monitor rheumatoid arthritis prevalence using hospital and medication databases. 2023. 10.1186/s13075-024-03366-x PMC11251372 39014427

[12] Tan JA , Roberts‐Thomson PJ , Blumbergs P , Hakendorf P , Cox SR , Limaye V . Incidence and prevalence of idiopathic inflammatory myopathies in South Australia: a 30‐year epidemiologic study of biopsy‐proven cases. Arthritis Rheum 2013; 63: 331–338. 10.1111/j.1756-185X.2011.01669.x 23981756

[13] Cann MP , Sage AM , McKinnon E , Lee SJ , Tunbridge D , Larkins NG *et al*. Childhood Systemic Lupus Erythematosus: presentation, management and long‐term outcomes in an Australian cohort. Lupus 2022; 31: 246–255. 35037500 10.1177/09612033211069765

[14] Eades LE , Sines J , Hoi AY , Liddle R , Kandane‐Rathnayake R , Morand EF *et al*. Autoimmune rheumatic disease in Australian Aboriginal and Torres Strait Islander Peoples: what do we know? Semin Arthritis Rheum 2024; 65: 152354. 38237231 10.1016/j.semarthrit.2023.152354

[15] Grennan DM , Bossingham D . Systemic lupus erythematosus (SLE): different prevalences in different populations of Australian aboriginals. Aust NZ J Med 1995; 25: 182–183. 10.1111/j.1445-5994.1995.tb02843.x 7605310

[16] Nigam A , Baer R , Green S , Neuen BL , Vile A , Mantha M . Lupus nephritis in Indigenous Australians: a single‐centre study. Intern Med J 2020; 50: 830–837. 31760686 10.1111/imj.14710

[17] Nossent J , Raymond W , Kang A , Wong D , Ongjenovic M , Chakera A . Impact of ethnicity on histology and outcome of lupus nephritis in western Australia. Intern Med J 2018; 48: 32.28782163

[18] Subramani P , Thomas S , Brady S , Pawar B , Cherian S , Fernandes K *et al*. A retrospective analysis on systemic lupus erythematosus in aboriginals and caucasians in central Australia. Nephrology 2016; 21: 207.

[19] Xu C , Goh KL , Abeyaratne A , Mogulla M , Majoni W , Priyadarshana K . Variations in clinical presentation and biomarkers among biopsy‐proven lupus nephritis patients: a Top End retrospective cohort study. Intern Med J 2023; 53: 531–539. 34697868 10.1111/imj.15596

[20] Englert H , Small‐McMahon J , Davis K , O'Connor H , Chambers P , Brooks P . Male systemic sclerosis and occupational silica exposure—A population‐based study. Aust NZ J Med 2000; 30: 215–220. 10.1111/j.1445-5994.2000.tb00810.x 10833113

[21] Munn Z , Moola S , Lisy K , Riitano D , Tufanaru C . Methodological guidance for systematic reviews of observational epidemiological studies reporting prevalence and cumulative incidence data. Int J Evid Based Healthc 2015; 13: 147–153. 26317388 10.1097/XEB.0000000000000054

[22] Liberati A , Altman DG , Tetzlaff J , Mulrow C , Gøtzsche PC , Ioannidis JP *et al*. The PRISMA statement for reporting systematic reviews and meta‐analyses of studies that evaluate health care interventions: explanation and elaboration. Ann Intern Med 2009; 151: W‐65–W‐94. 10.7326/0003-4819-151-4-200908180-00136 19622512

[23] New‐Tolley J , Smith C , Koszyca B , Otto S , Maundrell A , Bardy P *et al*. Inflammatory myopathies after allogeneic stem cell transplantation. Muscle Nerve 2018; 58: 790–795. 30194844 10.1002/mus.26341

[24] Lim JR , Nielsen TC , Dale RC , Jones HF , Beech A , Nassar N *et al*. Prevalence of autoimmune conditions in pregnant women in a tertiary maternity hospital: a cross‐sectional survey and maternity database review. Obstet Med 2021; 14: 158–163. 34646344 10.1177/1753495X20964680 PMC8504298

[25] Cronin O , Flanagan E , Dowling D . Prevalence and risk factors for the presence of autoimmune disease in an Australian cohort of patients with celiac disease. J Gastroenterol Hepatol 2018; 33: 120–121.

[26] Safiri S , Kolahi AA , Hoy D , Smith E , Bettampadi D , Mansournia MA *et al*. Global, regional and national burden of rheumatoid arthritis 1990–2017: a systematic analysis of the Global Burden of Disease study 2017. Ann Rheum Dis 2019; 78: 1463–1471. 31511227 10.1136/annrheumdis-2019-215920

[27] Ackerman IN , Bohensky MA , Pratt C , Gorelik A , Liew D . Counting the cost. Part 1 healthcare costs: the current and future burden of arthritis. 2016.

[28] Nossent JC , Keen HI , Preen DB , Inderjeeth CA . Population‐wide long‐term study of incidence, renal failure, and mortality rates for lupus nephritis. Int J Rheum Dis 2024; 27: e15079. 38396352 10.1111/1756-185X.15079

[29] Nossent J , Raymond W , Kang A , Wong D , Ognjenovic M , Chakera A . The current role for clinical and renal histological findings as predictor for outcome in Australian patients with lupus nephritis. Lupus 2018; 27: 1838–1846. 30092734 10.1177/0961203318792361

[30] Innovation VH . Covidence systematic review software Melbourne. 2024. www.covidence.org.

[31] Hoy D , Brooks P , Woolf A , Blyth F , March L , Bain C *et al*. Assessing risk of bias in prevalence studies: modification of an existing tool and evidence of interrater agreement. J Clin Epidemiol 2012; 65: 934–939. 22742910 10.1016/j.jclinepi.2011.11.014

[32] Barker TH , Migliavaca CB , Stein C , Colpani V , Falavigna M , Aromataris E *et al*. Conducting proportional meta‐analysis in different types of systematic reviews: a guide for synthesisers of evidence. BMC Med Res Methodol 2021; 21: 1–9. 34544368 10.1186/s12874-021-01381-z PMC8451728

[33] Migliavaca CB , Stein C , Colpani V , Barker TH , Ziegelmann PK , Munn Z *et al*. Meta‐analysis of prevalence: I 2 statistic and how to deal with heterogeneity. Res Synth Methods 2022; 13: 363–367. 35088937 10.1002/jrsm.1547

[34] Granholm A , Alhazzani W , Møller MH . Use of the GRADE approach in systematic reviews and guidelines. Br J Anaesth 2019; 123: 554–559. 31558313 10.1016/j.bja.2019.08.015

[35] Nossent J , Raymond W , Keen H , Inderjeeth C , Preen DB . Hospitalisation rates and characteristics for adult and childhood immunoglobulin A vasculitis in Western Australia. Intern Med J 2019; 49: 475–481. 30091295 10.1111/imj.14065

[36] Chung E , Risi D , Holt J , Lonergan M , Kotwal S , Yong K *et al*. A retrospective study on the epidemiology of ANCA‐associated vasculitis in two Australian health districts. Nephrology 2020; 25: 47. 10.1111/imj.15098 33040456

[37] Ormerod AS , Cook MC . Epidemiology of primary systemic vasculitis in the Australian Capital Territory and south‐eastern New South Wales. Intern Med J 2008; 38: 816–823. 18771432 10.1111/j.1445-5994.2008.01672.x

[38] Chan J , Aljishi M , Han T , Pham T , Ranganathan D , Borg J *et al*. Effect of severe cyclone on incidence and clinical phenotype of anti‐neutrophil cytoplasmic antibodies (ANCA) associated renal vasculitis (AAV) in central Queensland of Australia. Nephrology 2020; 25: 24.

[39] Pham T , Aljishi M , Chan J , Han T , McGrail M , Borg J *et al*. Effect of severe cyclone on incidence of glomerular disease in central Queensland of Australia. Nephrology 2020; 25: 25.

[40] Thet Z , Han T , Francis L , Aung N , Chau K , Ng SK *et al*. Epidemiology of glomerular diseases in the pre‐ and post‐COVID‐19 era in central Queensland, Australia. Nephrol Dial Transplant 2023; 38: i380.

[41] Oakman G , Bach CA , Ong C . Incidence of anti‐neutrophil cytoplasmic antibody‐associated renal vasculitis: a retrospective study in rural and regional Victoria, Australia. Intern Med J 2024; 54: 461–466. 37183767 10.1111/imj.16127

[42] Gray J , Soden M . Epidemiology of ANCA‐associated vasculitis in Townsville, North Queensland. Intern Med J 2015; 45: 20.

[43] Chau K , Ng SK , Lam A , Khoo T , Ranganathan D . Epidemiology of pauci‐immune ANCA‐associated glomerulonephritis – report from an Australian population‐based study. Nephrol Dial Transplant 2023; 38: i373.

[44] Tan PG , Whale K , Challis D , Unwin C , Kirkland G , Jeffs L *et al*., eds. Epidemiology of renal anti neutrophil cytoplasmic antibody (ANCA) associated vasculitis in Tasmania – A 10 year study. In: Rheumatology. Oxford, England: Oxford Univ Press; 2017.

[45] Paramalingam S , Raymond W , Sharma C , Dogra G , Mclean‐Tooke A , Nossent J . Disease flares, damage accrual and survival in ANCA‐associated vasculitis in Western Australia. Int J Clin Rheumatol 2019; 14: 31–36.

[46] Redondo‐Rodriguez R , Mena‐Vázquez N , Cabezas‐Lucena AM , Manrique‐Arija S , Mucientes A , Fernández‐Nebro A . Systematic review and metaanalysis of worldwide incidence and prevalence of antineutrophil cytoplasmic antibody (ANCA) associated vasculitis. J Clin Med 2022; 11: 2573. 35566698 10.3390/jcm11092573 PMC9106044

[47] Mackie FE , Kainer G , Adib N , Boros C , Elliott EJ , Fahy R *et al*. The national incidence and clinical picture of SLE in children in Australia – a report from the Australian Paediatric Surveillance Unit. Lupus 2015; 24: 66–73. 25288030 10.1177/0961203314552118

[48] Statistics ABo . Estimates of Aboriginal and Torres Strait Islander Australians 2021. Updated 30 June 2021. Available from: https://www.abs.gov.au/statistics/people/aboriginal-and-torres-strait-islander-peoples/estimates-aboriginal-and-torres-strait-islander-australians/latest-release.

[49] Eades LE , Hoi AY , Liddle R , Sines J , Kandane‐Rathnayake R , Khetan S *et al*. Systemic lupus erythematosus in Aboriginal and Torres Strait Islander peoples in Australia: addressing disparities and barriers to optimising patient care. Lancet Rheumatol 2024; 6: e713–e726. 38971169 10.1016/S2665-9913(24)00095-X

[50] Ong C , Nicholls K , Becker G . Ethnicity and lupus nephritis: an Australian single centre study. Intern Med J 2011; 41: 270–278. 21426464 10.1111/j.1445-5994.2009.02159.x

[51] Jegatheesan D , Nath K , Reyaldeen R , Sivasuthan G , John GT , Francis L *et al*. Epidemiology of biopsy‐proven glomerulonephritis in Queensland adults. Nephrology 2016; 21: 28–34. 26154936 10.1111/nep.12559

[52] Ghazanfari F , Jabbar Z , Nossent J . Renal histology in Indigenous Australians with lupus nephritis. Int J Rheum Dis 2018; 21: 194–199. 28762647 10.1111/1756-185X.13147

[53] Australian Bureau of Statistics . National Health Survey: Summary of Results 2007–2008. 2009.

[54] Australian Bureau of Statistics . National Health Survey 2022. 2023.

[55] Australian Bureau of Statistics . National Health Survey. 2022.

[56] Hill CL , Parsons J , Taylor A , Leach G . Health related quality of life in a population sample with arthritis. J Rheumatol 1999; 26: 2029–2035. 10493687

[57] Almutairi K , Inderjeeth C , Preen DB , Keen H , Nossent J . The prevalence of rheumatoid arthritis in Western Australia. BMC Rheumatol 2022; 6: 1–8. 36585680 10.1186/s41927-022-00324-5 PMC9804946

[58] Almutairi K , Nossent J , Preen DB , Keen H , Inderjeeth C . The temporal association between hospital admissions, biological therapy usage and direct health care costs in rheumatoid arthritis patients. Rheumatol Int 2022; 42: 2027–2037. 34536090 10.1007/s00296-021-04985-2

[59] González‐Chica DA , Vanlint S , Hoon E , Stocks N . Epidemiology of arthritis, chronic back pain, gout, osteoporosis, spondyloarthropathies and rheumatoid arthritis among 1.5 million patients in Australian general practice: NPS MedicineWise MedicineInsight dataset. BMC Musculoskelet Disord 2018; 19: 1–10. 29347932 10.1186/s12891-018-1941-x PMC5774097

[60] Bellamy N , Duffy D , Martin N , Mathews J . Rheumatoid arthritis in twins: a study of aetiopathogenesis based on the Australian Twin Registry. Ann Rheum Dis 1992; 51: 588–593. 1616321 10.1136/ard.51.5.588 PMC1005687

[61] Englert H , Small‐McMahon J , Davis K , O'Connor H , Chambers P , Brooks P . Systemic sclerosis prevalence and mortality in Sydney 1974–88. Aust NZ J Med 1999; 29: 42–50. 10.1111/j.1445-5994.1999.tb01587.x 10200812

[62] Roberts‐Thomson P , Jones M , Hakendorf P , Kencana Dharmapatni A , Walker J , MacFarlane J *et al*. Scleroderma in South Australia: epidemiological observations of possible pathogenic significance. Intern Med J 2001; 31: 220–229. 11456035 10.1046/j.1445-5994.2001.00048.x

[63] Roberts‐Thomson PJ , Walker JG , Lu TT , Esterman A , Hakendorf P , Smith MD *et al*. Scleroderma in South Australia: further epidemiological observations supporting a stochastic explanation. Intern Med J 2006; 36: 489–497. 16866652 10.1111/j.1445-5994.2006.01125.x

[64] Zochling J , Lewis P , Byron J , Walsh J , Dwyer N , Kilpatrick D . Why is there a high prevalence of scleroderma and secondary pulmonary hypertension in Tasmania, Australia? Intern Med J 2010; 40: 184–185.

[65] Zochling J , Lewis P , Morley P , Byron J , Stephens W , Dwyer N *et al*. Scleroderma: the TASSiE experience. Intern Med J 2009; 39: A62.

[66] Weisz N , Myat L , Abbot S , Ferns J , Reilly P . A review of patients with systemic sclerosis attending cairns Hospital: rates of decline in pulmonary function. Intern Med J 2017; 47: 34.

[67] Tian J , Kang S , Zhang D , Huang Y , Zhao M , Gui X *et al*. Global, regional, and national incidence and prevalence of systemic sclerosis. Clin Immunol 2023; 248: 109267. 36804224 10.1016/j.clim.2023.109267

[68] Khoo T , Lilleker JB , Thong BY‐H , Leclair V , Lamb JA , Chinoy H . Epidemiology of the idiopathic inflammatory myopathies. Nat Rev Rheumatol 2023; 19: 695–712. 37803078 10.1038/s41584-023-01033-0

[69] Giannini M , Debrut L , Nespola B , Velten M , Geny B , Sibilia J *et al*. Current classification criteria underestimate the incidence of idiopathic inflammatory myopathies by ignoring subgroups. Nat Rev Rheumatol 2024; 20: 311–312. 38514811 10.1038/s41584-024-01105-9

[70] Patrick M , Buchbinder R , Jolley D , Dennett X , Buchanan R . Incidence of inflammatory myopathies in Victoria, Australia, and evidence of spatial clustering. J Rheumatol 1999; 26: 1094–1100. 10332974

[71] Nossent J , Keen H , Preen DB , Inderjeeth CA . The spectrum of idiopathic inflammatory myopathies in Western Australia: epidemiological characteristics and mortality over time. Rheumatol Int 2024; 44: 329–337. 37819456 10.1007/s00296-023-05475-3 PMC10796655

[72] Needham M , Corbett A , Day T , Christiansen F , Fabian V , Mastaglia FL . Prevalence of sporadic inclusion body myositis and factors contributing to delayed diagnosis. J Clin Neurosci 2008;15: 1350–1353. 18815046 10.1016/j.jocn.2008.01.011

[73] Phillips BA , Zilko PJ , Mastaglia FL . Prevalence of sporadic inclusion body myositis in Western Australia. Muscle Nerve 2000; 23: 970–972. 10842277 10.1002/(sici)1097-4598(200006)23:6<970::aid-mus20>3.0.co;2-i

[74] Dobloug G , Antal E , Sveberg L , Garen T , Bitter H , Stjärne J *et al*. High prevalence of inclusion body myositis in Norway; a population‐based clinical epidemiology study. Eur J Neurol 2015; 22: 672–e41. 25530508 10.1111/ene.12627

[75] Nossent J , Inderjeeth C , Keen H , Preen D , Li I , Kelty E . The association between TNF inhibitor therapy availability and hospital admission rates for patients with ankylosing spondylitis. A longitudinal population‐based study. Rheumatol Ther 2022; 9: 127–137. 34762289 10.1007/s40744-021-00393-x PMC8814256

[76] Minaur N , Sawyers S , Parker J , Darmawan J . Rheumatic disease in an Australian Aboriginal community in North Queensland, Australia. A WHO‐ILAR COPCORD survey. J Rheumatol 2004; 31: 965–972. 15124258

[77] Stolwijk C , van Onna M , Boonen A , van Tubergen A . Global prevalence of spondyloarthritis: a systematic review and meta‐regression analysis. Arthritis Care Res 2016; 68: 1320–1331. 10.1002/acr.22831 26713432

[78] Lembke S , Macfarlane GJ , Jones GT . The worldwide prevalence of psoriatic arthritis—a systematic review and meta‐analysis. Rheumatology 2024; 63: keae198. 10.1093/rheumatology/keae198 PMC11637478 38530786

[79] Health AIo, Welfare . Medication Use for Ankylosing Spondylitis, Psoriatic Arthritis, and Juvenile Arthritis 2016–17. Canberra: AIHW; 2019.

[80] Tian J , Zhang D , Yao X , Huang Y , Lu Q . Global epidemiology of systemic lupus erythematosus: a comprehensive systematic analysis and modelling study. Ann Rheum Dis 2023; 82: 351–356. 36241363 10.1136/ard-2022-223035 PMC9933169

[81] Izmirly PM , Parton H , Wang L , McCune WJ , Lim SS , Drenkard C *et al*. Prevalence of systemic lupus erythematosus in the United States: estimates from a meta‐analysis of the Centers for Disease Control and Prevention National Lupus Registries. Arthritis Rheumatol 2021; 73: 991–996. 33474834 10.1002/art.41632 PMC8169527

[82] Rees F , Doherty M , Grainge M , Davenport G , Lanyon P , Zhang W . The incidence and prevalence of systemic lupus erythematosus in the UK, 1999–2012. Ann Rheum Dis 2016; 75: 136–141. 25265938 10.1136/annrheumdis-2014-206334 PMC4717400

[83] Ellis J , McHugh N , Pauling JD , Bruce IN , Charlton R , McGrogan A *et al*. Changes in the incidence and prevalence of systemic lupus erythematosus between 1990 and 2020: an observational study using the Clinical Practice Research Datalink (CPRD). Lupus Sci Med 2024; 11: e001213. 39067871 10.1136/lupus-2024-001213 PMC11284910

[84] Eades LE , Sines J , Hoi AY , Liddle R , Kandane‐Rathnayake R , Morand EF *et al*., eds. Autoimmune Rheumatic Diseases in Australian Aboriginal and Torres Strait Islander Peoples: what do we know? In: Seminars in Arthritis and Rheumatism. Philadelphia: Elsevier; 2024. 10.1016/j.semarthrit.2023.152354 38237231

[85] Sines J , Cai K , Cashman B , Abbott P , Zengin A , Manolios N *et al*. The burden of rheumatologic disease in Aboriginal and Torres Strait Islander Australians. Intern Med J 2024; 54: 1603–1615. 39136359 10.1111/imj.16489

[86] Almutairi K , Inderjeeth C , Preen D , Keen H , Nossent J . The prevalence of rheumatoid arthritis in Western Australia. Int J Rheum Dis 2023; 26: 92–93. 10.1186/s41927-022-00324-5 PMC9804946 36585680

[87] Nossent J , Raymond W , Isobel Keen H , Preen D , Inderjeeth C . Morbidity and mortality in adult‐onset IgA vasculitis: a long‐term population‐based cohort study. Rheumatology 2022; 61: 291–298. 10.1093/rheumatology/keab312 33779729

[88] Almutairi K , Nossent J , Preen D , Keen H , Inderjeeth C . The global prevalence of rheumatoid arthritis: a meta‐analysis based on a systematic review. Rheumatol Int 2021; 41: 863–877. 33175207 10.1007/s00296-020-04731-0

[89] Chung CP , Rohan P , Krishnaswami S , McPheeters ML . A systematic review of validated methods for identifying patients with rheumatoid arthritis using administrative or claims data. Vaccine 2013; 31: K41–K61. 24331074 10.1016/j.vaccine.2013.03.075

[90] Moores KG , Sathe NA . A systematic review of validated methods for identifying systemic lupus erythematosus (SLE) using administrative or claims data. Vaccine 2013; 31: K62–K73. 24331075 10.1016/j.vaccine.2013.06.104

[91] Lee H , Ford JA , Jin Y , Cho SK , Santiago Ortiz AJ , Tong AY *et al*. Validation of claims‐based algorithms for psoriatic arthritis. Pharmacoepidemiol Drug Saf 2020; 29: 404–408. 31849154 10.1002/pds.4950

[92] Eder L , Widdifield J , Rosen CF , Alhusayen R , Cheng SY , Young J *et al*. Identifying and characterizing psoriasis and psoriatic arthritis patients in Ontario administrative data: a population‐based study from 1991 to 2015. J Rheumatol 2020; 47: 1644–1651. 32062600 10.3899/jrheum.190659

[93] Gau SY , Tsai HE , Wang YH , Wei JCC . Validation of diagnosis algorithms for ankylosing spondylitis in claim‐based database. Int J Rheum Dis 2024; 27: e15041. 38287537 10.1111/1756-185X.15041

[94] Sreih AG , Annapureddy N , Springer J , Casey G , Byram K , Cruz A *et al*. Development and validation of case‐finding algorithms for the identification of patients with anti‐neutrophil cytoplasmic antibody‐associated vasculitis in large healthcare administrative databases. Pharmacoepidemiol Drug Saf 2016; 25: 1368–1374. 27804171 10.1002/pds.4116 PMC5135635

[95] Hannah JR , Gordon PA , Galloway J , Rutter M , Peach EJ , Rooney M *et al*. Validation of methods to identify people with idiopathic inflammatory myopathies using hospital episode statistics. Rheumatol Adv Pract 2022; 6: rkac102. 36532317 10.1093/rap/rkac102 PMC9749128

[96] Almutairi K , Inderjeeth C , Preen DB , Keen H , Rogers K , Nossent J . The accuracy of administrative health data for identifying patients with rheumatoid arthritis: a retrospective validation study using medical records in Western Australia. Rheumatol Int 2021; 41: 741–750. 33620516 10.1007/s00296-021-04811-9

[97] Nossent J , Raymond W , Ognjenovic M , Kang A , Wong D , Chakera A . The role for renal histology as predictor for outcome in lupus nephritis in Western Australia. Int J Rheum Dis 2018; 21: 164.

[98] Kitching AR , Anders H‐J , Basu N , Brouwer E , Gordon J , Jayne DR *et al*. ANCA‐associated vasculitis. Nat Rev Dis Primers 2020; 6: 71. 32855422 10.1038/s41572-020-0204-y

[99] Dirikgil E , Tas SW , Verburgh CA , Soonawala D , Hak AE , Remmelts HH *et al*. Identifying relevant determinants of in‐hospital time to diagnosis for ANCA‐associated vasculitis patients. Rheumatol Adv Pract 2022; 6: rkac045. 35784016 10.1093/rap/rkac045 PMC9245319

[100] Hunter RW , Welsh N , Farrah TE , Gallacher PJ , Dhaun N . ANCA associated vasculitis. BMJ 2020; 369: m1070. 32291255 10.1136/bmj.m1070 PMC7179255

[101] Limaye V , Blumbergs P , Scott G , Hakendorf P , Stevanovic V , Highton J *et al*. The epidemiology of dermatomyositis in South Australia. APLAR J Rheumatol 2007; 10: 94–100.

[102] Lundberg IE , Tjärnlund A , Bottai M , Werth VP , Pilkington C , de Visser M *et al*. EULAR/ACR classification criteria for adult and juvenile idiopathic inflammatory myopathies and their major subgroups. Ann Rheum Dis 2017; 76: 1955.29079590

[103] Day J , Patel S , Limaye V , eds. The role of magnetic resonance imaging techniques in evaluation and management of the idiopathic inflammatory myopathies. In: Seminars in Arthritis and Rheumatism. Philadelphia: Elsevier; 2017. 10.1016/j.semarthrit.2016.11.001 28088340

[104] Luu Q , Day J , Hall A , Limaye V , Major G . External validation and evaluation of adding MRI or extended myositis antibody panel to the 2017 EULAR/ACR myositis classification criteria. ACR Open Rheumatol 2019; 1: 462–468. 31777826 10.1002/acr2.11061 PMC6858041

[105] Parker MJ , Kim PS , Beer K , Panniker A , Fong G , Needham M . Rationale, objectives and design of a national prospective database for idiopathic inflammatory myopathies: the Australian Myositis Registry. Intern Med J 2024; 55: 47–56. 39614828 10.1111/imj.16593

[106] Muntyanu A , Milan R , Rahme E , LaChance A , Ouchene L , Cormier M *et al*. Exposure to silica and systemic sclerosis: a retrospective cohort study based on the Canadian Scleroderma Research Group. Front Med 2022; 9: 984907. 10.3389/fmed.2022.984907 PMC9556811 36250083

[107] Patel S , Morrisroe K , Proudman S , Hansen D , Sahhar J , Sim MR *et al*. Occupational silica exposure in an Australian systemic sclerosis cohort. Rheumatology 2020; 59: 3900–3905. 32911541 10.1093/rheumatology/keaa446

[108] Mehri F , Jenabi E , Bashirian S , Shahna FG , Khazaei S . The association between occupational exposure to silica and risk of developing rheumatoid arthritis: a meta‐analysis. Saf Health Work 2020; 11: 136–142. 32596007 10.1016/j.shaw.2020.02.001 PMC7303526

[109] Gómez‐Puerta JA , Gedmintas L , Costenbader KH . The association between silica exposure and development of ANCA‐associated vasculitis: systematic review and meta‐analysis. Autoimmun Rev 2013; 12: 1129–1135. 23820041 10.1016/j.autrev.2013.06.016 PMC4086751

[110] Muntyanu A , Milan R , Rahme E , Baron M , Netchiporouk E , Baron M *et al*. Organic solvent exposure and systemic sclerosis: a retrospective cohort study based on the Canadian Scleroderma Research Group registry. J Am Acad Dermatol 2023 3: 605–607. 10.1016/j.jaad.2023.04.062 37182702

[111] Opinc‐Rosiak AH , Makowska JS . Environmental exposures as risk factors for idiopathic inflammatory myopathies. J Autoimmun 2023; 140: 103095. 37797402 10.1016/j.jaut.2023.103095

[112] Darikwa TB , Manda SO . Measuring Bivariate Spatial Clustering in Disease Risks. In: Modern Biostatistical Methods for Evidence‐Based Global Health Research. Cham: Springer; 2022; 235–260.

[113] Gómez‐Rubio V , López‐Quílez A . Statistical methods for the geographical analysis of rare diseases. Rare Dis Epidemiol 2010; 686: 151–171. 10.1007/978-90-481-9485-8_10 20824445

[114] Zochling J , Lewis P , Byron J , Langsford D , Walsh J , Dwyer N *et al*. The epidemiology of scleroderma and secondary pulmonary hypertension in Tasmania, Australia. Clin Exp Rheumatol 2010; 28: S130.

