# Supplementary material for: Prevalence of vasculitis, systemic lupus erythematosus, rheumatoid arthritis, systemic sclerosis, idiopathic inflammatory myopathies and spondyloarthritis in Australia: a systematic review and meta‐analysis
Source: Intern Med J. 2025 Sep 15;55(12):1985–96. doi: 10.1111/imj.70210 (PMC12704404; doi:10.1111/imj.70210)
Supplement: Supplementary file 1 — Data S1: Supporting Information. [file IMJ-55-1985-s001.docx]

***Supplementary material:* Prevalence of Autoimmune Rheumatic Diseases in Australia: A Systematic Review and Meta-Analysis**

**1. SEARCH STRATEGY**

| **Example search terms (full search strategy below)** | | | | | | | |
| --- | --- | --- | --- | --- | --- | --- | --- |
| **Disease** | |  | **Setting** |  | | **Study purpose** | |
| **Combined with OR**:  systemic lupus erythematosus, scleroderma, systemic sclerosis, vasculitis, myositis, idiopathic inflammatory myopath*, rheumatoid arthritis, psoriatic arthritis, ankylosing spondylitis, spondyloarth*, behcet*, Sjogren* | | **AND** | **Combined with OR**:  Australia, New South Wales, Queensland, Victoria*, Western Australia*, Tasmania* , South Australia* | **AND** | | **Combined with OR:**  incidence, prevalence, rate, surveillance, population-based, disease adj3 pattern, disease adj3 burden | |
| **Full search strategy** | | | | | | | |
| **Database** | **Diseases** | | | | **Study type** | | **Context** |
| **Medline** | Lupus  SLE  Rheumatoid arthritis  Psoriatic arthritis  Vasculitis  *Myositis myopathy, dermatomyositis, polymyositis  Scleroderma  Systemic sclerosis  Sjogren*  Behcet*  Spondylo*  Ankylosing spond* | | | | Incidence  Prevalence  Epidemiology  Rate  Disease adj3 burden  Disease adj3 pattern  Surveillance  Population-Based | | Australia* Western Aus*  Tasmania*  Victoria*  South Austra*  Queensland  New South Wales NSW  Northern Territory*  Australian Capital Territory |
| **MeSH terms to. Use (within medline)** | Lupus erythematosus, Systemic  Arthritis, rheumatoid  Arthritis, psoriatic  Anti-Neutrophil Cytoplasmic Antibody-Associated vasculitis  IgA vasculitis  Systemic vasculitis  Vasculitis | | | | Incidence  Prevalence  Epidemiology  Population Surveillance | | Australia  South Australia  Western Australia  New South Wales  Queensland  Tasmania  Victoria  Northern Territory  Australian Capital Territory |
| **Embase terms** | Lupus erythematosus  Systemic lupus erythematosus  Rheumatoid arthritis  Psoriatic arthritis  Vasculitis  ANCA associated vasculitis  Small vessel vasculitis  Large vessel vasculitis  Systemic vasculitis  Myositis  Inclusion body myositis  Sporadic inclusion body myositis  Idiopathic inflammatory myopathy  Dermatomyositis  Polymyositis  Scleroderma  Diffuse scleroderma  Limited scleroderma  Systemic sclerosis  Sjoegren syndrome [spelt this way within EMBASE term headings]  Behcet disease  Spondyloarthropathy  Ankylosing spondylitis | | | | Incidence, cumulative incidence  prevalence, period prevalence, point prevalence | |  |
| **CINAHL headings** | Lupus erythematosus, Systemic  As above. | | | | Prevalence  Epidemiology  Incidence  Disease hotspot | | Australia Western Australia  South Australia  First Nations of Australia |
| **SCOPUS** | Lupus erythematosus OR "Systemic lupus erythematosus" OR "Rheumatoid arthritis" OR "Psoriatic arthritis" OR Vasculitis OR "ANCA associated vasculitis" OR "Small vessel vasculitis" OR "Large vessel vasculitis" OR "Systemic vasculitis"  OR  Myositis OR "Inclusion body myositis" OR "Sporadic inclusion body myositis" OR "Idiopathic inflammatory myopathy" OR Dermatomyositis OR Polymyositis OR "Scleroderma" OR "Diffuse scleroderma" OR "Limited scleroderma" OR "Systemic sclerosis" OR "Sjogren syndrome" OR "Behcet disease" OR Spondyloarthropathy OR "Ankylosing spondylitis" | | | | Prevalence, epidemiology, incidence  Burden w/3 disease  Pattern w/3 disease | | Australia* Western Aus*  Tasmania*  Victoria*  South Austra*  Queensland  New South Wales NSW  Northern Territory  Australian Capital Territory |
| **Web of science** (no thesauraus for scopus or web of science hence search is the same) |  |  |  |  | Prevalence, epidemiology, incidence  Burden near/3 disease  Pattern near/3 disease | |  |
| **Google scholar** | Lupus erythematosus OR "Systemic lupus erythematosus" OR "Rheumatoid arthritis" OR "Psoriatic arthritis" OR Vasculitis OR "ANCA associated vasculitis" OR "Small vessel vasculitis" OR "Large vessel vasculitis" OR "Systemic vasculitis"  [new search as there is word limit per search in google]  Myositis OR "Inclusion body myositis" OR "Sporadic inclusion body myositis" OR "Idiopathic inflammatory myopathy" OR Dermatomyositis OR Polymyositis OR "Scleroderma" OR "Diffuse scleroderma" OR "Limited scleroderma" OR "Systemic sclerosis" OR "Sjogren syndrome" OR "Behcet disease" OR Spondyloarthropathy OR "Ankylosing spondylitis" | | | | Prevalence, epidemiology, incidence | | Australia |
| **Google** | Lupus erythematosus OR "Systemic lupus erythematosus" OR "Rheumatoid arthritis" OR "Psoriatic arthritis" OR Vasculitis OR "ANCA associated vasculitis" OR "Small vessel vasculitis" OR "Large vessel vasculitis" OR "Systemic vasculitis"  [new search as there is word limit per search in google]  Myositis OR "Inclusion body myositis" OR "Sporadic inclusion body myositis" OR "Idiopathic inflammatory myopathy" OR Dermatomyositis OR Polymyositis OR "Scleroderma" OR "Diffuse scleroderma" OR "Limited scleroderma" OR "Systemic sclerosis" OR "Sjogren syndrome" OR "Behcet disease" OR Spondyloarthropathy OR "Ankylosing spondylitis" | | | | Prevalence, epidemiology, incidence | | Australia  Gov  Pdf |

**2. VASCULITIS**

**2.1 Vasculitis Study Characteristics**

| **Vasculitis type** | **Study** | **Region** | **Period** | **Case ascertainment** | **Disease definition** | **Data (I/P)** | **Bias** |
| --- | --- | --- | --- | --- | --- | --- | --- |
| ANCA + IgA | Thet 2023 | QLD | 2017 - 2022 | Renal bx database: central QLD | NR | I | High |
| ANCA | Chan 2020 | QLD | 2012-2018 | NR | NR | I | High |
|  | Pham 2020 | QLD | 2012 - 2018 | NR | NR | I | High |
|  | Gray 2015 | QLD | 2009 - 2013 | All positive ANCA results at Townsville Hospital. | ACR criteria GPA or Chapel Hill criteria MPA/EGPA | I | Low |
|  | Paramalingam 2019 | WA | 2007 - 2016 | Discharge diagnoses and all outpatient rheum/immuno/nephrology clinic letters searched for 'vasculitis'; single hospital. | EMA (Europeans Medicine Agency) algorithm | I/P | Low |
|  | Chau 2023 | QLD | 2005 - 2021 | Renal bx database: Royal Brisbane and Women's Hospital | 212 Chapel Hill Consensus Conference definition | I | Mod |
|  | Tan 2017 | TAS | 2004 - 2013 | Renal bx databse: Tas | | I | Low |
|  | Chung 2020 | NSW | 2002 - 2017 | Electronic medical record review of hospital data from ISLHD and SESLHD | NR | I | Mod-high |
|  | Ormerod 2008 | ACT and surrounds | 1995 - 2004 | Discharge diagnosis codes for two hospitals; all ANCA at one hospital lab. | ACR 1990 criteria GPA, PAN, EGPA, confirmed with record review | I/P | Low |
|  | Oakman 2024 | VIC | 2013 - 2021 | Renal bx database: Bendigo Health | 2022 ACR/EULAR criteria | I | Mod |
| GPA | Hissaria 2008 | SA | 2001-2005 | Clinical databases of renal, rheumatology, respiratory units and two large SA hospitals, all positive ANCA at main laboratory service | ACR 1990 criteria for GPA | I | Low |
| Takayasu | Makin 2016 | WA | 2000 - 2015 | Discharge diagnoses and outpatient communication were searched for keywords related to TAK at three hospitals in WA | ACR 1990 Classification Criteria for TAK | I/P | Low |
| IgA | Nossent 2019 [1] | WA | 1980 - 2015 | Inpatient and ED discharge diagnosis codes; all WA hospitals. | ICD-10 codes | I | Low |
| Giant Cell Arteritis | Ninan 2023 | SA | 2014 - 2020 | All temporal artery biopsies at SA Pathology labs (reviews ~75% of SA specimens) | Pathologist diagnosis from temporal artery biopsy | I | Low |
|  | Dunstan 2014 | SA | 1991 - 2011 | All temporal artery biopsy from three labs (~85% of SA specimens) | Pathologist diagnosis on report. | I | Low |
| Unclear | Briganti 2001 | VIC | 1995 – 1997 | Renal bx database | “Vasculitis” – unclear subtype | I | High |
| *ISLHD, Illawara Shoalhaven Local Health District; SESLHD, South East Sydney Local Health District; TAK, Takayasu Arteritis; IgA, IgA vasculitis; ACR, American College of Rheumatology; GPA, Granulomatosus with polyangiitis. | | | | | | | |

**2.2 Vasculitis Study Findings**

| **Vasculitis type** | **Study** | **Population denominator** | **Total cases^** | **Reported prevalence per million** | **Included in Meta-Analysis** |
| --- | --- | --- | --- | --- | --- |
| ANCA | Paramalingam 2019 | WA North Metropolitan Health Service: 540,000 | 63 | 116.7 | No |
|  | Ormerod 2008 | ACT and Queanbeyan, Southern Tablelands and Snowy Mtn regions: 448,000 | 74 | 148.4 | No |
| Takayasu | Makin 2016 | All of WA: 2.4 million | 13 | 3.9 | No |
|  |  |  |  | **Reported incidence / million person years** | |
| ANCA + IgA | Thet 2023 | Central Queensland Health Service: 220,912 | 13 | AAV: 4.5 – 15 (pre/post COVID)  IgAN: 18 – 4.5 (pre/post COVID) | No |
| ANCA | Chan 2020 | QLD - Central Queensland health district: 230,000 | 11 | 1.5 to 15 (pre/post cyclone) | No |
|  | Pham 2020 | QLD - Central Queensland health district: 230,000 | 11 | 1.5 – 15 (pre/post cyclone) | No |
|  | Gray 2015 | Townsville Health District: 230,000 | 14 | 12.2^ | Yes |
|  | Paramalingam 2019 | WA North Metropolitan Health Service: 540,000 | 63 | 10.1 | Yes |
|  | Chau 2023 | Royal Brisbane and Women's Hospital Catchment: 1.03 million | 80 | 4.6 | Yes |
|  | Tan 2017 | All of Tas: 500,000 | 61 | 11.9 | Yes |
|  | Chung 2020 | SESLHD, ISLHD: 600,000 | 156 | 6.84 (SESLHD), 12.28 (ISLHD) | Yes |
|  | Ormerod 2008 | ACT and Queanbeyan, Southern Tablelands and Snowy Mtn regions: 448,000 | 74 | 12.4 (ACT), 25.9 (rural NSW)* | Yes |
|  | Oakman 2024 | Loddon Mallee Public Health Unit: 340,000 | 28 | 9.3 | Yes |
| GPA | Hissaria 2008 | All of SA: 1.5 million | 84 | 11.2 | No - GPA alone |
| IgA | Nossent 2019 | All of WA: 2.5 million | 620 | 25.9 (<20yo), 3.3 (adult) | No |
| Giant Cell Arteritis | Ninan 2023 | All of SA: 1.7 million | 181 | 54 | No |
|  | Dunstan 2014 | All of SA: 1.5 million | 314 | 32 | No |
| Various | Briganti 2001 | All of Vic: 4.6 million | 2030 | 12 to 19 | No |

**2.3 Vasculitis: Forest Plot**

The forest plot displays the pooled incidence rates for ANCA-associated vasculitis using log-transformed data and a random-effects model. The methods used included log transformations for small rates, standard error calculations, and back-transformation of results for interpretability.


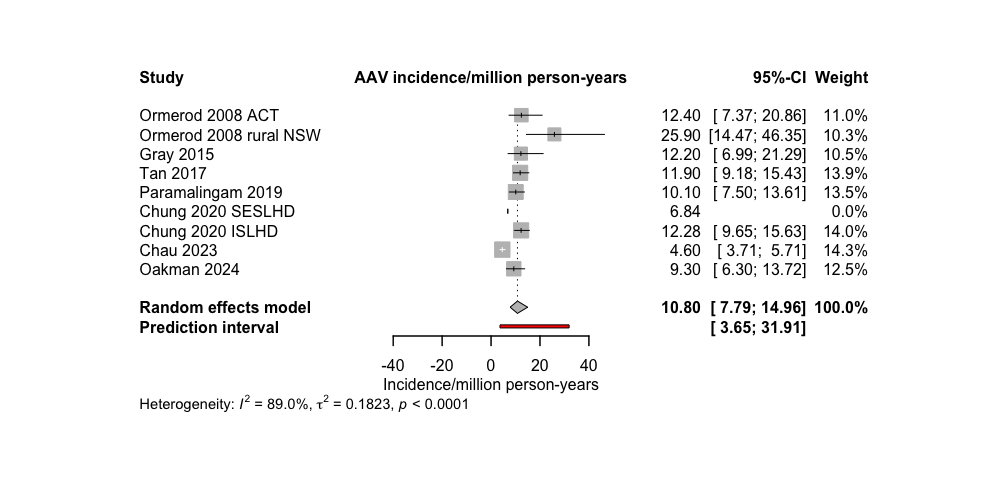


**3. SYSTEMIC LUPUS ERYTHEMATOSUS**

**3.1 Systemic Lupus Erythematosus and Lupus Nephritis Study Characteristics**

| **Disease** | **Study** | **Region** | **Period** | **Case ascertainment** | **Disease definition** | **Data (P/I)** | **Risk of Bias** |
| --- | --- | --- | --- | --- | --- | --- | --- |
| **Systemic Lupus Erythemat-osus** | Cann 2022 | WA | 1998-2018 | Discharge and outpatient records of Perth Children's hospital Rheumatology department were screened. All children with SLE in WA are referred to this hospital. | 2012 SLICC criteria | I | Mod |
|  | Subramani 2017 | NT^ | 1999 - 2016 | Alice Springs Hospital discharge diagnosis of SLE. | 2012 SLICC criteria | P | Mod |
|  | Mackie 2015 | Australia | 2009 - 2011 | Aus paediatric surveillance unit (APSU) = monthly mail to paediatricians actively recruiting SLE patients. | >4/11 ACR criteria (definite), ANA>1:320 and one ACR (probable) | I | Mod |
|  | Bossingham 2003 | QLD | 1996 - 1998 | All patients seen by the only rheumatologist in the region (private, public hospital and clinics) + physicians, GP s and staff of Aboriginal health services were contacted for further case finding | 1982 ARA criteria | P | Mod |
|  | Segasothy 2001 | NT^ | 1990 - 1999 | All GPs, DMOs and nephrologists in central Australia were contacted. | 1997 ACR criteria for SLE | P | Mod |
|  | Grennan 1995 | Australia | 1993-1994 | All rheumatologist and dermatologists and three Aboriginal health centres in Sydney, and renal physicians at RNS hospital, were contacted for SLE patient details. One rheumatologist (referred most patients in Cairns, and provided all remote outreach) provided QLD data. | NR | P | High |
|  | Anstey 1993 | NT | 1984 - 1991 | All positive ANAs >1:40 at Darwin/Katherine Hospital, contacted all physicians in Top End. | 1982 American Rheumatism Association criteria for SLE | I/P | Mod-High |
| **Lupus Nephritis** | Nossent 2024 | WA | 1985 - 2015 | >2 hospital discharge codes for SLE and diagnostic codes for AKI, dialysis, renal biopsy or transplant | ICD-10 codes | I/P | Low |
|  | Nossent 2018 [2] | WA | 1997 - 2017 | Renal biopsy database from a single centre | Specific histological criteria | I/P* | Low |
|  | Briganti 2001 | VIC | 1995-1997 | Renal biopsy database: Vic | Not specified | I* | Mod |
|  | Xu 2023^^ | NT | 2010 - 2019 | Renal biopsy database for Top End of the NT | Specific histological criteria | I* | Mod |
|  | Ghazanfari 2018^^ | NT | 2001 - 2011 | Renal biopsy database: Royal Darwin Hospital | Specific histological criteria | I* | Mod |
|  | Ong 2011 | VIC | 2005-2007 | Renal biopsy database from a single centre | Specific histological criteria | P | Low |
|  | Jegatheesan 2016 | QLD | 2002 - 2011 | Renal biopsy database: QLD | Specific histological criteria | I* | Mod |
| *confidence interval for incidence not provided calculated for modelling, assuming Poisson distribution.  ^Overlapping populations: Alice Springs Hospital catchment/Central Australia  ^^Overlapping populations: renal biopsy database Top End of NT, renal biopsy database Royal Darwin Hospital (for which the catchment is the entire Top End of the NT). | | | | | | | |

**3.2 Systemic Lupus Erythematosus and Lupus Nephritis Study Findings**

| **Disease** | **Study** | **Population size** | **Cases identified** | **Incidence/100,000 person-years (95% confidence interval)** | **Prevalence /100,000** | **Included in meta-analysis** |
| --- | --- | --- | --- | --- | --- | --- |
| **Systemic Lupus Erythemat-osus** | Cann 2022 | 550000 | 42 (88%); Aboriginal 26.2%; Asian 23.8% | 0.44* | NA | No |
|  | Subramani 2017^ | 18631 (Aboriginal), 32,408 (non-Aboriginal | 31 (aboriginal), 39 (non-aboriginal) | NA | 166 Aboriginal, 25 non-Aboriginal | Yes |
|  | Mackie 2015 | 4.2 million | 30 | 0.35* | NA | No |
|  | Bossingham 2003 | 28,000 (Aboriginal)  210,000 (non-Aboriginal) | 26 (Aborigina), 82 (non-Aboriginal) | NA | 92 Aboriginal, 39 non-Aboriginal | Yes |
|  | Segasothy 2001^ | 19,000 (Aboriginal), 31,000 (non-Aboriginal) | 6 (non-Aboriginal), 14 (Aboriginal) | NA | 74 Aboriginal, 19 non-Aboriginal | Yes |
|  | Grennan 1995 | 22,400 (QLD), 22,905 (NSW) | 20 (QLD), 3 (NSW) | NA | 89 QLD, 13 NSW | No |
|  | Anstey 1993 | 24900 | 13 | NA | 52 | Yes |
| **Lupus Nephritis** | Nossent 2024 [3] | 2.5 million | 366 | 0.67 (0.42, 1.08) | 11.9 | Yes |
|  | Nossent 2018 [2] | 600,000 | 90 | Total: 0.75. Asian: 3.3. Aboriginal: 3.1. | 15 (total), 65.3 (Asian), 61.1 (Aboriginal) | Yes |
|  | Briganti 2001 | All of Victoria | 203 | 0.9 (M), 2.6 (F)* | NA | No |
|  | Xu 2023^^ | 177,500 | 33 | 5.08 (Aboriginal) 0.47 (non-Aboriginal)* | NA | Yes |
|  | Ghazanfari 2018^^ | 177,500 | 45 | 2.1 (total), 7 (Aboriginal), 0.7 (non-Aboriginal)* | NA | Yes |
|  | Ong 2011 [4] | 2.6 million | 145 | NA | 5.5 (total), 27.9 (Asian) | Yes |
|  | Jegatheesan 2016 | 3.4 million | 213 | 0.69 | NA | Yes |

*confidence interval for incidence not provided calculated for modelling, assuming Poisson distribution.

^ Overlapping populations: Alice Springs Hospital catchment/Central Australia

^^Overlapping populations: renal biopsy database Top End of NT, renal biopsy database Royal Darwin Hospital (for which the catchment is the entire Top End of the NT).

**3.3 Systemic Lupus Erythematosus Prevalence: Forest Plot**

The forest plot shows the pooled prevalence of SLE using the double arcsine transformation and a random-effects model, with back-transformation of results for interpretability. Two studies—Subramani (2017) and Segasothy (2001)—reported on patients from Central Australia but sampled from non-overlapping time periods (1999–2011 and 1990–1999, respectively) and used distinct case identification methods (hospital discharge diagnoses versus direct contact with local clinicians). As both studies represent the same geographic region, we assessed whether their inclusion might disproportionately influence the pooled estimate. To explore this, each study was removed in turn. The pooled prevalence with both included was 57.86 per 100,000 (95% CI: 33.12–89.19), which increased to 65.32 when Segasothy was excluded and decreased to 50.66 when Subramani was excluded. As the two studies shifted the pooled result in opposite directions, both were retained in the final meta-analysis.

The final model, including four studies, was also analysed by subgroup due to its wide prediction interval, demonstrating higher prevalence of SLE in Aboriginal and Torres Strait Islander people than non-Aboriginal Australians. Importantly, the pooled prevalence estimate is applicable only to Northern Australia, as all included studies were conducted in regions with a higher proportion of Aboriginal and Torres Strait Islander peoples than the national average.


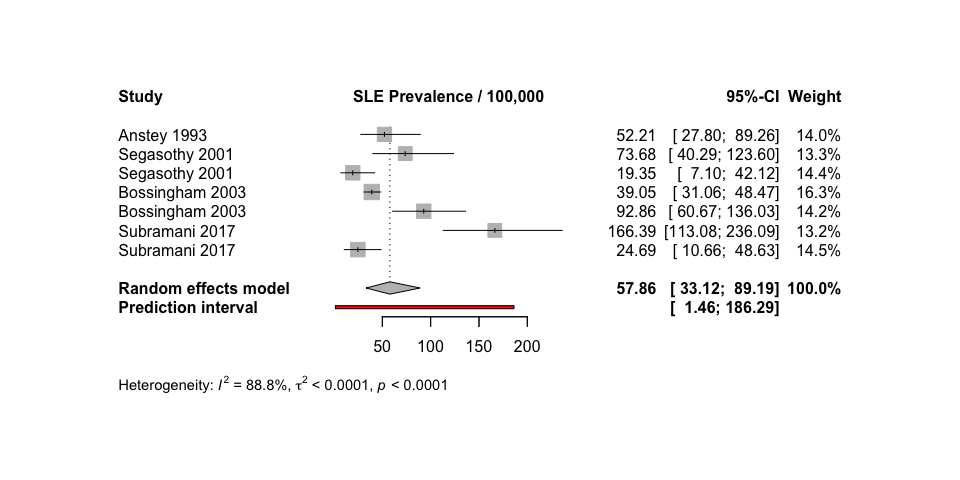


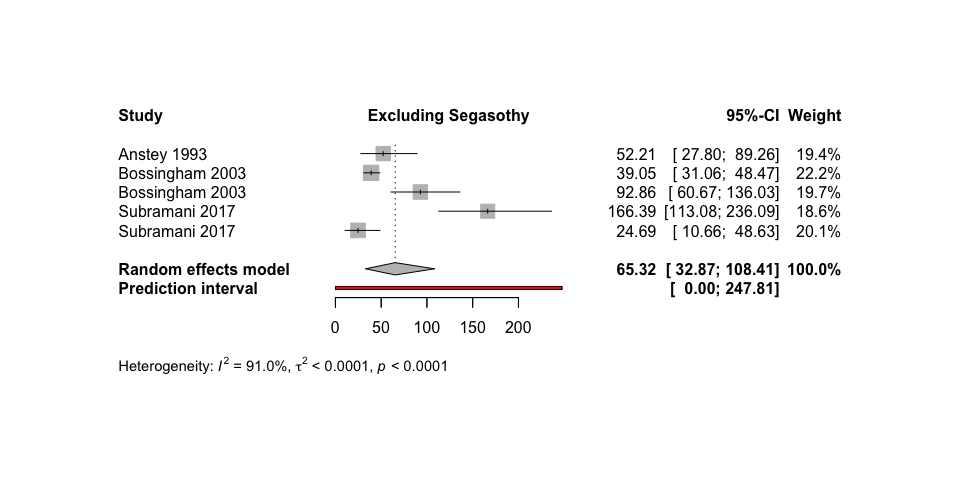

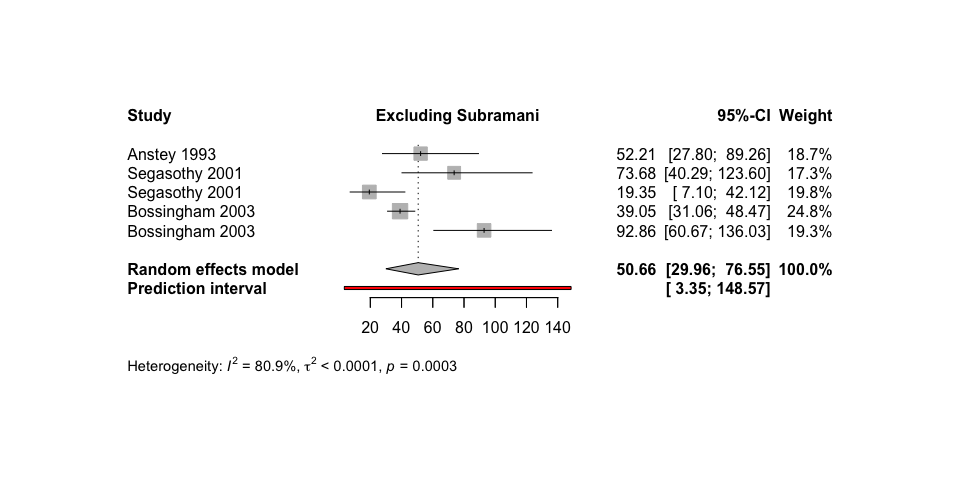


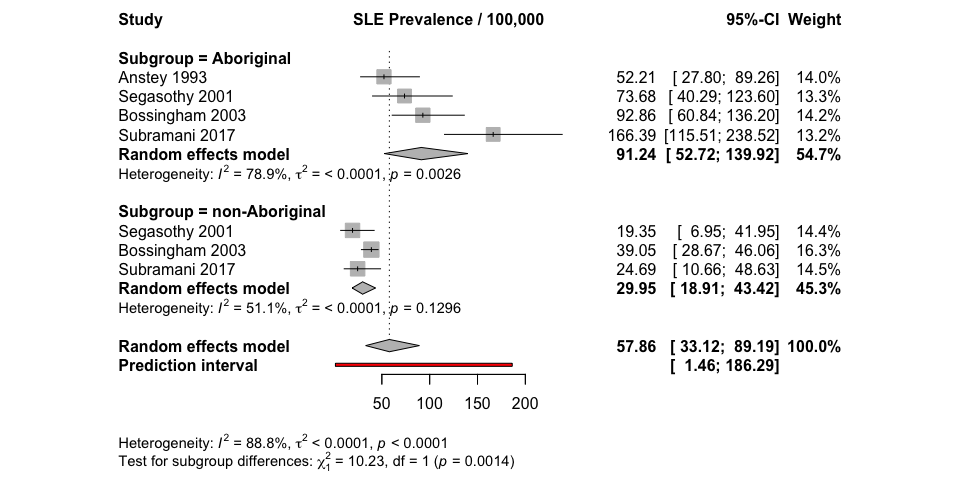


**3.4 Lupus Nephritis Prevalence: Forest Plot**

The forest plot illustrates the pooled prevalence of Lupus Nephritis, using the double arcsine transformation and a random-effects model, with back-transformation of pooled results for interpretability.


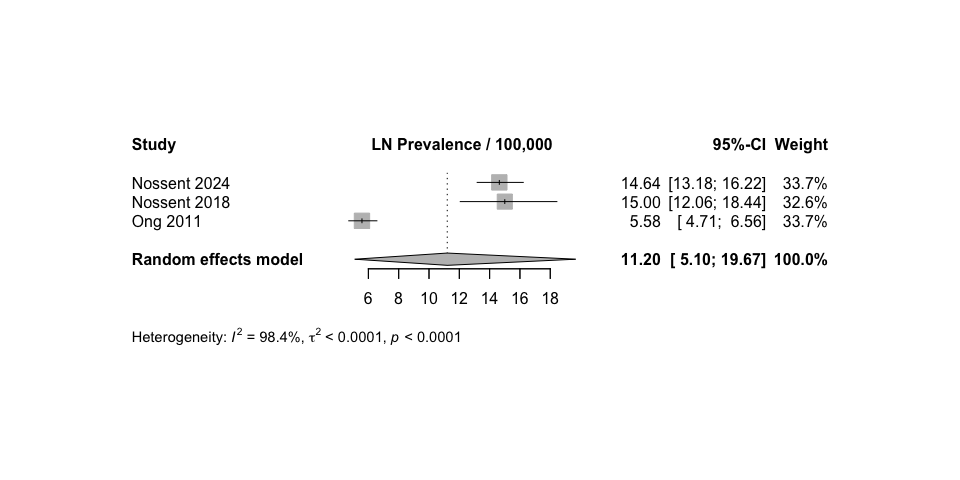


**3.5 Lupus Nephritis Incidence: Forest Plot**

The forest plot illustrates the pooled incidence of Lupus Nephritis using a random-effects model and log-transformed data. Two studies—Ghazanfari (2018) and Xu (2023)—were conducted over sequential time periods (2001–2011 and 2010–2019, respectively), but both may include overlapping populations from the Top End of the Northern Territory, as they drew on renal biopsy data from similar sources (Top End Renal Biopsy Database and Royal Darwin Hospital records). To assess whether this geographic concentration biased the overall estimate, we conducted sensitivity analyses including both studies, Xu alone, and Ghazanfari alone. The pooled annual incidence rate (AIR) with both studies included was 1.25 per 100,000 person-years (95% CI: 0.55–2.85). Removing Xu yielded an AIR of 1.13, while removing Ghazanfari yielded 1.02. Both studies shifted the estimate in the same direction, with overlapping confidence intervals and a change of <2 per 100,000, but the relative difference exceeded 10%. Therefore, only the more recent study (Xu) was retained in the final meta-analysis.

Given the wide prediction interval, we also conducted subgroup analyses using the three studies—Nossent (2018), Xu (2023), and Ghazanfari (2018)—that reported incidence separately for Aboriginal and Torres Strait Islander peoples. These analyses confirmed a markedly higher incidence of LN among Aboriginal and Torres Strait Islander peoples compared to non-Indigenous Australians (5.11 vs. 0.47 per 100,000 person-years). However, as these estimates derive from only three studies across two geographic regions, they should be interpreted as evidence of differential incidence, rather than as representative pooled estimates of national incidence for each group.


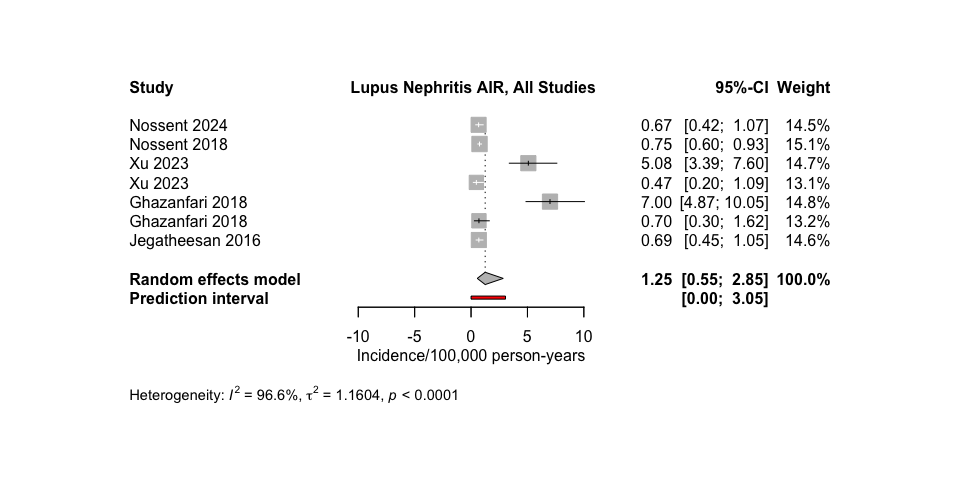

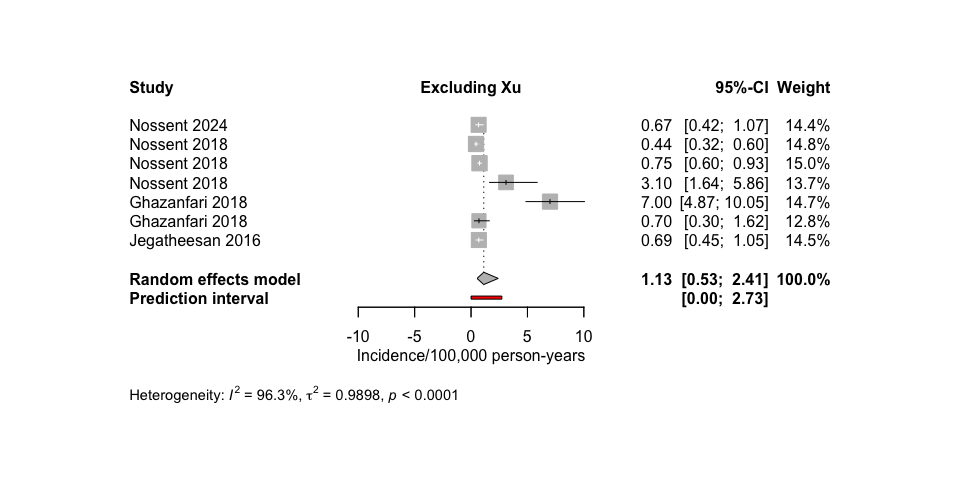


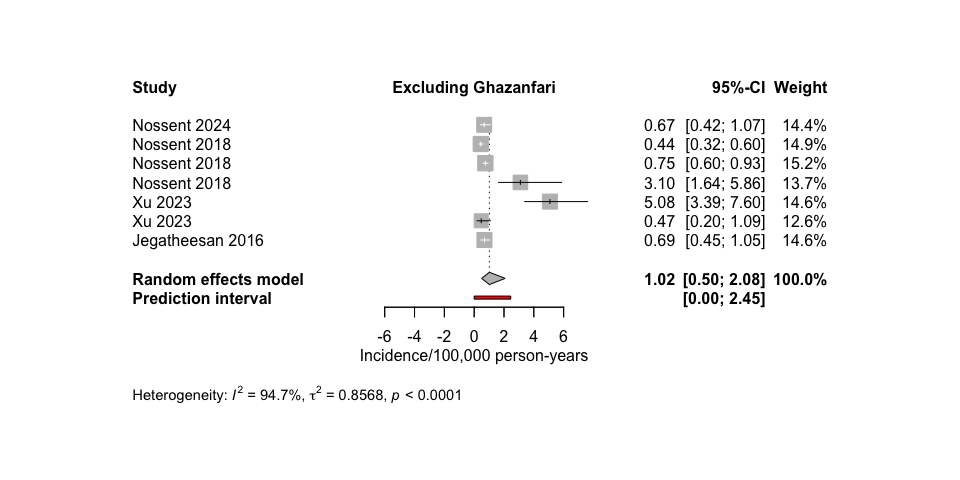


The forest plot demonstrates the subgroup analysis for Lupus Nephritis AIR between Aboriginal and non-Aboriginal populations.


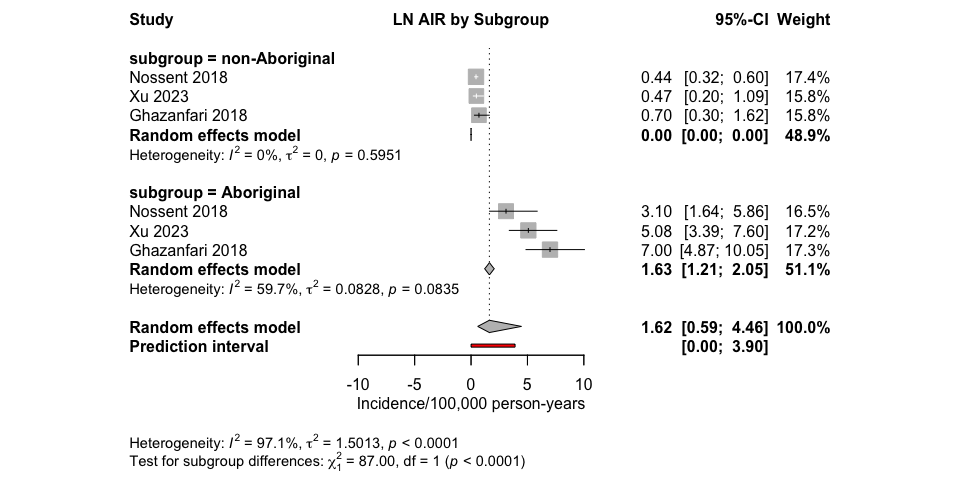


**4. RHEUMATOID ARTHRITIS**

**4.1 Rheumatoid Arthritis Study Characteristics**

| **Study** | **Region** | **Period** | **Case ascertainment** | **Disease definition** | **Data (P/I)** | **Bias** |
| --- | --- | --- | --- | --- | --- | --- |
| ABS 2023 [5] | Australia | 2022 | In person interviews of stratified multistage area sample of private dwellings | Self-report | p | High: self-report |
| Koller-Smith 2023[6] | Australia | 2005 - 2015 | Linked ALSWH, PBS, hospital, ED, MBS data. Five methods of case ascertainment compared: self-report, PBS dispensing of RA medications, ICD-10 codes for RA, self-report of RA specific medications, and the above corrected for hosp/PBS codes suggesting an alternate autoimmune condition | Self-report, PBS dispensing, ICD-10 codes | P | Low: but applicable to Australian Women only - 100% female population |
| Almutairi 2022 [7] | WA | 1995 - 2014 | Hosp codes for RA, used for prevalence/1000 separations. Annual dispensings of RA bDMARDS multiplied by 2 (assumed 50% of RA patients use biologics) used to calculate period prevalence. | Hospital codes, receiving bDMARD for RA | P | Mod-High |
| Gonzalez-Chica 2018 [8] | Australia | 2000 - 2016 | GP entered diagnosis of RA or spondyloarthropathy into their electronic medical records (MedicineInsight database). | GP diagnosis | P | Low-mod:  Based on unverified entry into routine electronic medical record |
| ABS 2015 [9] | Australia | 2014-2015 | In person interviews of stratified multistage area sample of private dwellings | Self-report | p | High: self-report |
| ABS 2012 | Australia | 2011 | In person interviews of stratified multistage area sample of private dwellings | Self-report | P | High: self-report |
| ABS 2009 [10] | Australia | 2007 - 2008 | In person interviews of stratified multistage area sample of private dwellings | Self-report | P | High: self-report |
| AIHW 2009 | Australia | 2004-2005 | In person interviews of stratified multistage area sample of private dwellings | Self-report | p | High: self-report |
| Hill 1999 [11] | SA | 1995 | In person interviews of systematic, clustered area sample of 4,200 households | Self-report | p | Mod |
| Bellamy 1992 [12] | Australia | 1978 - 1982 | Surveys posted to twins on the Australian NHMRC twin registry | 1987 ARA criteria for the classification of RA | P | Low |

*Insufficient data for confidence interval calculation provided

**4.2 Rheumatoid Arthritis Study Findings**

|  | | | |  |
| --- | --- | --- | --- | --- |
| **Study** | **Population denominator** | **Cases** | **Prevalence (%)** | **Included in meta-analysis:** |
| ABS 2023 [5] | NR | NR | 2 | No |
| Koller-Smith 2023[6] | ALSWH: 25,467 | 292 - 1367 | Self-report: 5.4% (3.9% corrected) Hospital data: 1.1% PBS data: 2.8% (1.9% corrected) Self-report medications: 1.5% (0.5% corrected) | No |
| Almutairi 2022 [7] | Annual WA population 2003 - 2014 | 17125 | Hospital data: 0.34%  Dispensing data: 0.36% (2005-09) – 0.72% (2010 – 2014) (corrected for 50% use assumption) | No |
| Gonzalez-Chica 2018 [8] | All Australians in the Medicine Insight dataset who were an active patient of a participating GP in 2000-2016 = 1,501,267 people | NR | 0.9% (0.8, 1) | No |
| ABS 2015 [9] | 19,259 | NR | 2.8 | No |
| ABS 2012 | NR | NR | 2 | No |
| ABS 2009 [10] | 25,900 | 518 | 2 | No |
| AIHW 2009 | Representative Australian sample: 20,788 | 436 | 2.1 | No |
| Hill 1999 [11] | Sampled households: 3,001 | 27 | 0.89* | No |
| Bellamy 1992 [12] | Australian NHMRC Twin Registry | 186 | 0.4 | No |

*Only abstract available. Reported as 666/3001 with any arthritis, and 4% of those with RA. RA prevalence calculated from this.

**5. SYSTEMIC SCLEROSIS**

**5.1 Systemic Sclerosis Study Characteristics**

| **Study** | **Location** | **Period** | **Case identification** | **Disease definition** | **Data (I/P)** | | **Risk of bias** |
| --- | --- | --- | --- | --- | --- | --- | --- |
| Abbot 2020^^ (Abbot, McWilliams et al. 2020) | QLD | 2018 | Cairns Hospital outpatient records + direct contact with nephrologists, gastroenterologists, respiratory physicians and cardiologists in Cairns who may have had SSc patients. | 2013 ACR/EULAR diagnostic criteria | | P | Low |
| Zochling 2010^†^ (Zochling, Lewis et al. 2010) | TAS | 2007-2009 | All GPs, rheumatologists and pulmonary hypertension clinics and SSc patient support groups in Tasmania were contacted. | Unspecified | | I/P | Mod |
| Zochling 2009^†^ (Zochling, Lewis et al. 2009) | TAS | 2007 | All GPs, rheumatologists and pulmonary hypertension clinics and SSc patient support groups in Tasmania were contacted | Unspecified | | I/P | Mod |
| Weisz 2017^^ (Weisz, Myat et al. 2017) | QLD | 1999 - 2016 | Cairns hospital rheumatology outpatient department records. | Unspecified | | P | Mod |
| Roberts-Thomson 2006 | SA | 1993-2002 | Analysis of the SA Scleroderma Registry (described below). | ARA 1980 criteria | | I/P | Low |
| Roberts-Thomson 2001 | SA | 1993-1999 | Creation of the SA Scleroderma Registry. Participants ascertained through multiple sources: Hospital discharge codes of major teaching hospitals; all positive centromere and Scl-70 in state labs; participation of all rheumatologists in SA; death data; patients self-referred; nailfold capillaroscopy clinic | ARA 1980 criteria | | P | Low |
| Chandran 1995 (Chandran, Smith et al. 1995) | SA | 1993 | Hospital discharge diagnosis codes from 5 hospitals, outpatient records of two hospitals, state death data was searched. | unspecified | | P | Mod |
| Englert 2005 (Englert, Joyner et al. 2005) | VIC | 1986 - 1996 | All SSc patients known to the single GP in the area. GPs in surrounding region were surveyed for additional | Rheumatologist diagnosis. | | P | Low-mod |
| Englert 1999 (Englert, Small-McMahon et al. 1999) | NSW | 1974 - 1988 | All members of Scleroderma NSW were sent an invitation. The two largest medical labs in NSW had all those with positive ANA screened. Discharge diagnoses from all public and large private hospitals in Sydney and outpatient data from 2 hospitals. State death data interrogated. Physicians, vascular surgeons and dermatologists with contact details listed by RACP were contacted. | ACR 1980 criteria | | P | Low |
| ^^Overlapping populations: Cairns.  ^†^Overlapping populations: Tasmania | | | | | | | |

**5.2 Systemic Sclerosis Study Findings**

| **Study** | **Population denominator** | **Cases** | **Prevalence (per 100,000)** | **Included in Meta-Analysis** |
| --- | --- | --- | --- | --- |
| Abbot 2020 | Cairns and surrounding regions: 240190 | 81 | 34 | Yes |
| Zochling 2010 | All of Tasmania: 493000 | 157 | 31 | Yes |
| Zochling 2009 | All of Tasmania: 493000 | 120 | 24 | Yes |
| Weisz 2017 | Cairns Hospital catchment: 160000 | 73 | 46 | Yes |
| Roberts-Thomson 2006 | All of SA: 1.46 - 1.52 million over study period | 321 | 21 | Yes |
| Roberts-Thomson 2001 | All of SA: 1.46 - 1.49 million over study period | 348 | 23 | Yes |
| Chandran 1995 | All of SA: 1.46 million | 304** | 20.8 | Yes |
| Englert 2005 | Edenhope region: 9830 | 6 | 61 | Yes |
| Englert 1999 | Population of Sydney 3.2 milion*. | 715 | 4.5-8.6^ | Yes |
| * Not verifiable as ABS no longer reports greater Sydney population using these boundaries. For inclusion in meta-analysis later measure (8.6) used.  **only 215 actual cases identified, extrapolated to be 304, as 3 hospitals did not provide outpatient data so their rates of outpatient SSc were assumed to match the other hospitals  ^ Total 715 identified over 14 years, with considerable mortality rate. Assumed 275 alive at time of period prevalence calculation, as annual mortality rate not available, and 275 is in keeping with reported prevalence proportion and population denominator  ^^Overlapping populations: Cairns.  ^†^Overlapping populations: Tasmania | | | | |

**5.3 Systemic Sclerosis Forest Plot**

The forest plot shows the pooled prevalence of SSc using the double arcsine transformation and a random-effects model, with back-transformation of pooled results for interpretability. The dotted line refers to the pooled prevalence estimate. To assess the impact of overlapping populations, a sensitivity analysis was conducted due to multiple studies from Cairns (n=2), Tasmania (n=2), and South Australia (n=3). Four models were compared:

(1) including only the largest study in each overlapping group (Abbot 2020 over Weisz 2017, Zochling 2010 over Zochling 2009, and Roberts-Thomson 2006 for South Australia) (prevalence 24.8/100,000);
(2) including only the smallest study in each group (Weisz 2017, Zochling 2009, and Chandran 1995) (prevalence 24.8/100,000);
(3) including Roberts-Thomson 2001 for South Australia (with Abbot 2020 and Zochling 2010 in the other groups) (prevalence 25.4/100,000); and
(4) including all overlapping studies (prevalence 25.6/100,000).

All four models produced estimates within 2 per 100,000, with <10% relative difference, and remained within the original 95% confidence interval. Accordingly, all studies were retained in the final meta-analysis.


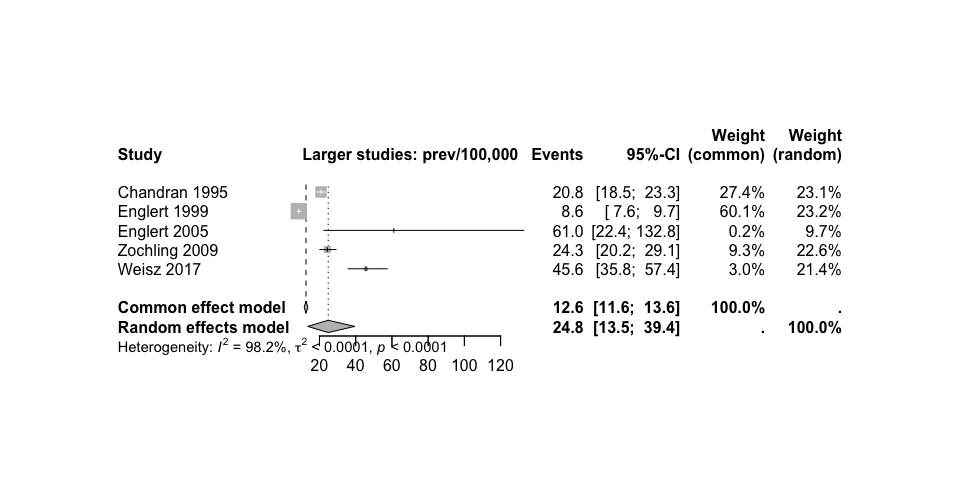


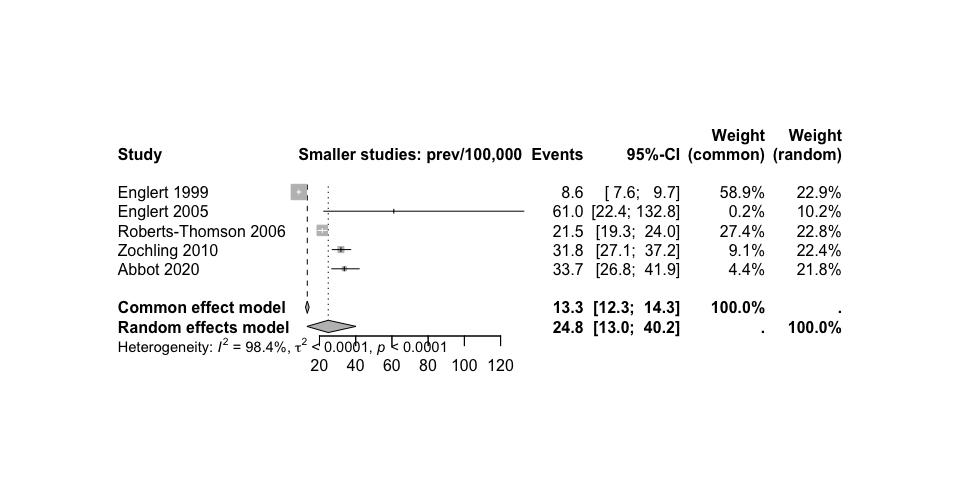


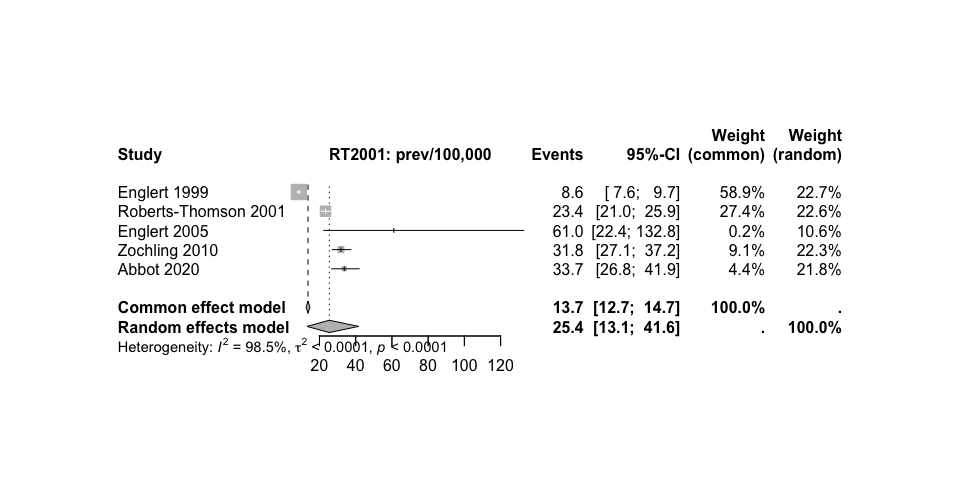


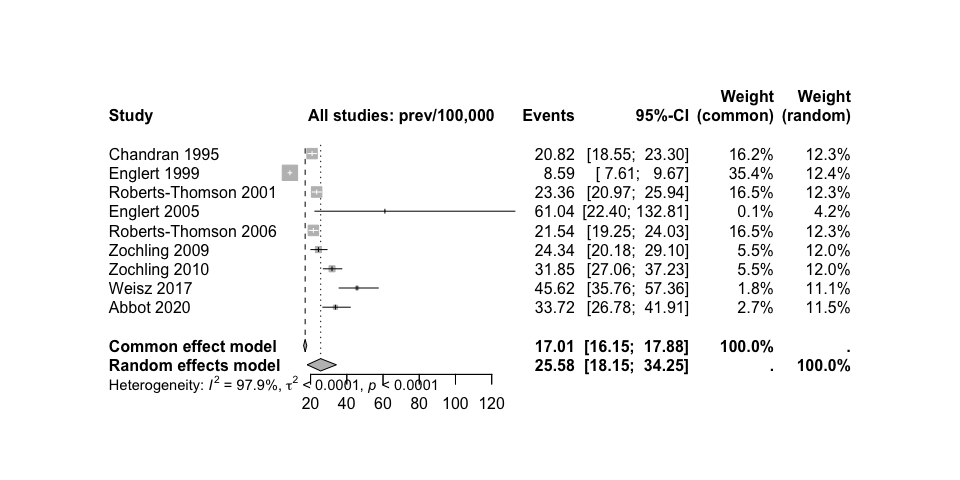


We also assessed the influence of the outlier result from the Englert 2005 study on the overall estimate. This study was designed to investigate a possible disease cluster in a small regional population and reported a much higher prevalence than other studies. The analysis showed that exclusion of Englert 2005, altered the pooled prevalence by less than 2 per 100,000. This reflects the relatively small weighting assigned to Englert 2005 by the random-effects model, due to its high variance and small population size.


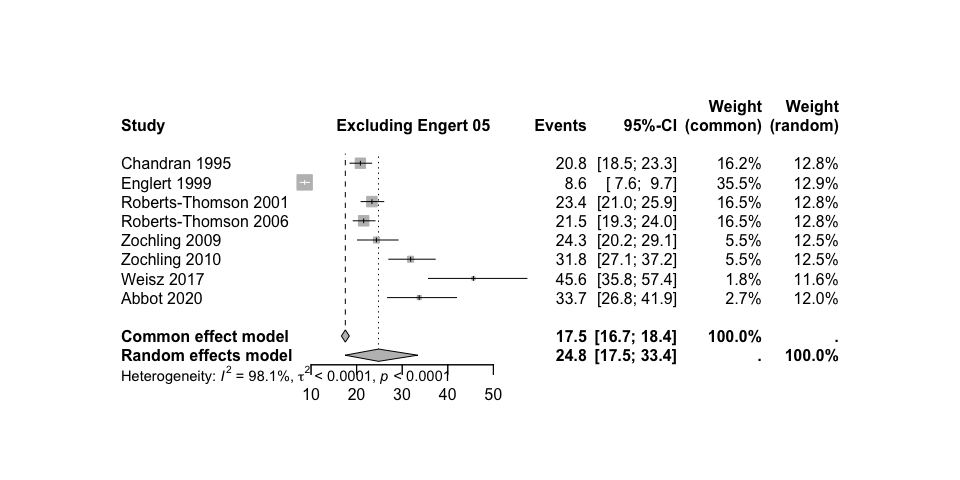


**6. IDIOPATHIC INFLAMMATORY MYOPATHIES**

**6.1 Idiopathic Inflammatory Myopathies Study Characteristics**

| **Study** | **Disease** | **State** | **Period** | **Case identification** | **Disease definition** | **Data (I/P)** | **Risk of bias** |
| --- | --- | --- | --- | --- | --- | --- | --- |
| Nossent 2024[10] | IIM - defined as DM, PM, IBM, other IIM, overlap myositis | WA | 1980-2014 | Hospital admission with myositis, using internationally validated algorithm of ICD10 codes for IIM. IBM identified using 359.7, 359.8 (pre 2000) then G72.4 (‘inflammatory myopathy, not elsewhere specified) from 2000-2014. | ≥1 hospital admission with ICD-10 code: for DM (M33.0, M33.1, M33.9), PM (M33.2), PM/DM (M36.0), inflammatory myopathy (G72.4). other myositis (M60.8) or myositis unspecified (M60.9) only if it occurred alongside an ILD code (J84.1, J84.9 or J99.1). Mapped to ICD-9 codes for pre-ICD10 era. | P (IBM, IIM), I (IBM, IIM, DM, PM) | Low-Mod |
| Tan 2013 [11] | IIM defined as DM, PM, IBM | SA | 1980 - 2009 | All muscle biopsies in the state. Medical records reviewed for correlation. Only patients fulfilling histological criteria DM/PM/IBM, >18yo, from SA included | Histological criteria as defined by authors | P (IBM), I (IBM, IIM, DM, PM) | Mod |
| Needham 2008 [12] | IBM | WA | 2007 | All patients of the Inflammatory Myopathies Clinic (Australian Neuromuscular Research Institute). All muscle biopsies in the state; letters sent to all neurologists and rheumatologists in WA | Clinical and biopsy criteria proposed by Needham and Mastaglia. Only definite and probable IBM included | P (IBM) | Low |
| Limaye 2007 [13] | IIM - defined as DM, PM | SA | 1990-2005 | All muscle biopsies in state; discharge diagnoses of all SA hospitals. But reported incidence is of biopsy proven IIM alone. | ICD 9-10 codes for PM, DM, juvenile DM, other DM, DM/PM unspecified. Or: muscle biopsy histological criteria described by author. | I (DM, PM) | Low |
| Patrick 1999 [14] | IIM (Peter and Bohan and including IBM), juvenile myosiits, overlap myositis, malignancy associated myositis | VIC | 1989 - 1991 | All muscle biopsies in state, linked to discharge diagnoses of 6 largest hospitals. | Biopsy: Banker and Engel criteria for PM and DM. Or discharge diagnoses: ICD-9 codes for PM, DM, myalgia-suspected myositis, inflammatory myopathy.  Cases identified by the above underwent clinical record review and included if met Bohan and Peter criteria for definite/probable IIM. | I (IIM, DM, PM) | Mod |
| Phillips 2000 [15] | IBM | WA | 1988 - 1998 | All patients of the Inflammatory Myopathies Clinic (Australian Neuromuscular Research Institute). All muscle biopsies from 3 metropolitan Perth hospitals, questionnaire sent to all rheumatologists and neurologists. | Griggs et al clinical and biopsy criteria - but MUST have biopsy criteria to be included. | P (IBM) | Low |

**6.2 Idiopathic Inflammatory Myopathies Study Findings**

| **Study** | **Population denominator** | **Cases** | **Prevalence per million** | **Included in pooled prevalence** |
| --- | --- | --- | --- | --- |
| Nossent 2024 [13] | 2.5 million | 146 (IBM), 847 (IIM)^ | 23.9 (IBM), 205 (IIM) | IBM: yes, IIM: no |
| Tan 2013 [14] | 1.5 million | 76 (IBM) | 50.5 | IBM: yes |
| Needham 2008 [15] | 2.08 million | 31 (IBM) | 14.9 (50.3 in over 50s) | IBM: yes |
| Phillips 2000 [16] | 1.8 million | 17 (IBM) | 9.3 | IBM: yes |
|  |  |  | **Incidence per million person years** | **Included in pooled incidence** |
| Nossent 2024[13] | 2.5 million | 847 (IIM overall) | 19 (IIM), 5.0 (DM), 7.3 (PM), 3.3 (IBM) | DM: yes, PM: yes, IIM, IBM: N/A |
| Tan 2013 [14] | 1.5 million | 352 (IIM overall) | 8.0 (IIM), 1.0 (DM), 4.1 (PM), 2.9 (IBM) | DM: yes, PM: yes, IIM, IBM: N/A |
| Limaye 2007 [17] | 1.5 million | 782 (DM + PM) | 2.9 (DM) (corrected), 6.6 (PM) | DM: yes, PM: yes, IIM, IBM: N/A |
| Patrick 1999 [18] | 4.42 million | 94 (IIM) | 7.4 (IIM), 1.2 (DM), 5.5 (PM) | DM: yes, PM: yes, IIM, IBM: N/A |
| ^Total 146 cases seen, with 101 deaths over 34 years. We assumed 59 alive at time of point prevalence calculation, to match the authors reported prevalence of 23.9/million and ABS reported population denominator | | | | |

**6.3 Idiopathic Inflammatory Myopathies Incidence**

The forest plot displays the pooled incidence rates for IIM using log-transformed data and a random-effects model (Limaye is not included as only reported PM and DM, not overall IIM). We conducted a sensitivity analysis excluding the outlier study Nossent (2024), which reduced the pooled incidence from 9.96 to 7.86 cases per million person-years. The higher estimate in Nossent is likely attributable to the use of ICD codes alone to identify IIM cases, without chart review for clinical confirmation—a limitation discussed in the manuscript. However, as Nossent provides the most contemporary data available, we chose to retain both pooled estimates in the manuscript and clearly discuss this potential limitation in the manuscript, for maximum transparency.


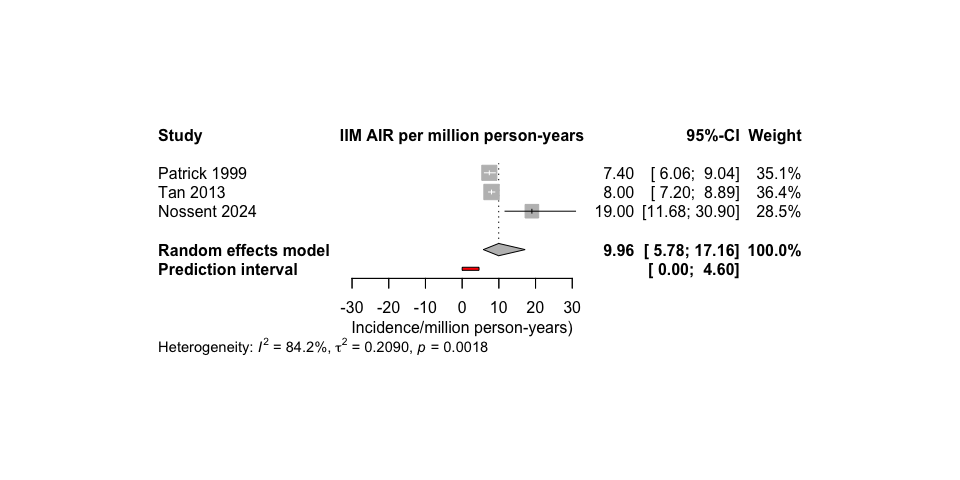


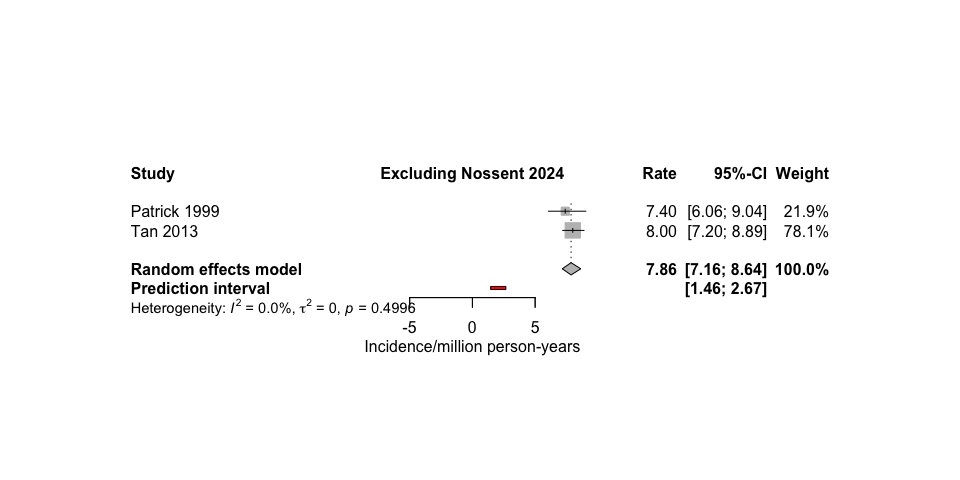


**6.4 Dermatomyositis Incidence**

The forest plot displays the pooled incidence rates for DM using log-transformed data and a random-effects model. Two studies—Tan and Limaye—included overlapping populations from the same region. To assess the influence of this region on the overall estimate, we conducted sensitivity analyses excluding each study in turn. The pooled annual incidence rate (AIR) was 1.77 per million person-years with both included, decreasing to 1.34 when Limaye was excluded and increasing to 2.90 when Tan was excluded. As the studies shifted the pooled estimate in opposite directions, both were retained in the meta-analysis.

This discrepancy likely reflects methodological differences: Limaye identified cases through hospital administrative data, potentially including individuals without biopsy confirmation, while Tan restricted inclusion to biopsy-proven cases. Additionally, the earlier time period in Tan’s study (1980–1990) may reflect lower disease recognition and diagnostic accuracy compared to the later period covered by Limaye (1990–2005), as discussed in the manuscript.


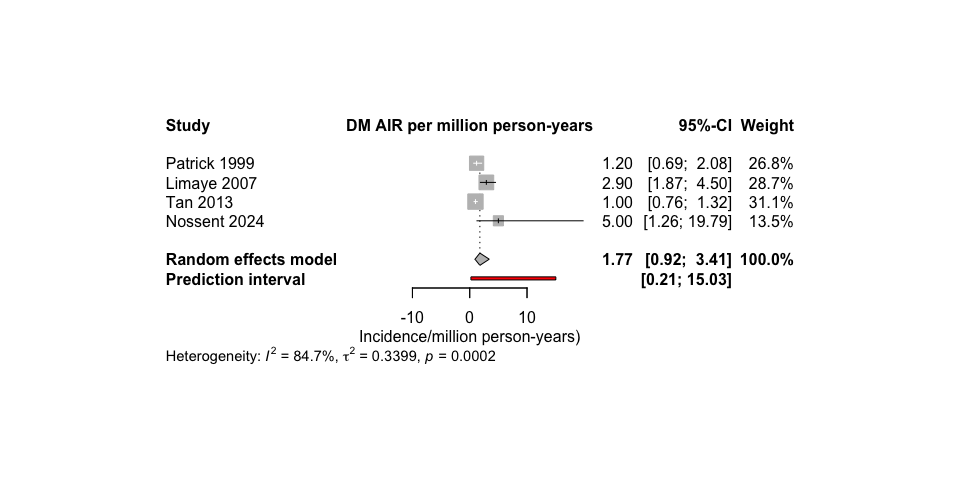


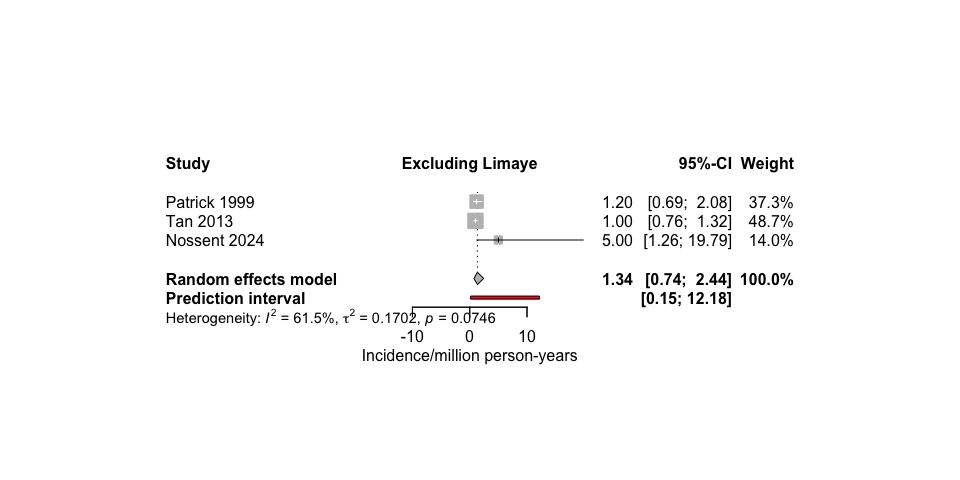


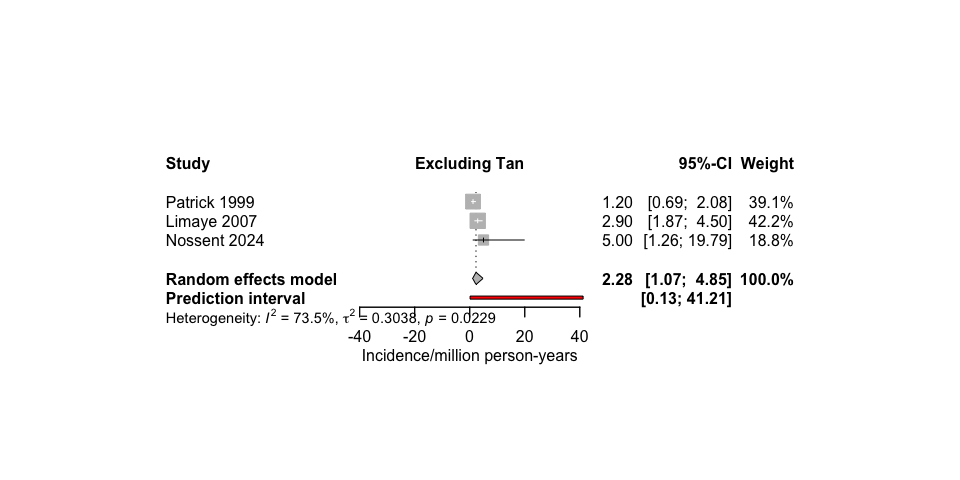


**6.5 Polymyositis Incidence**

The forest plot displays the pooled incidence rates for PM using log-transformed data and a random-effects model. As for dermatomyositis, the polymyositis studies included two from overlapping populations but used different case-finding methods and had slightly different time periods (Tan and Limaye). The AIR estimate with all studies was 5.18 (95% CI: 3.98–6.75) per million person-years. Removing Tan increased the estimate to 5.87, while removing Limaye decreased it to 4.82. As the studies shifted the pooled estimate in opposite directions, both were retained in the meta-analysis.


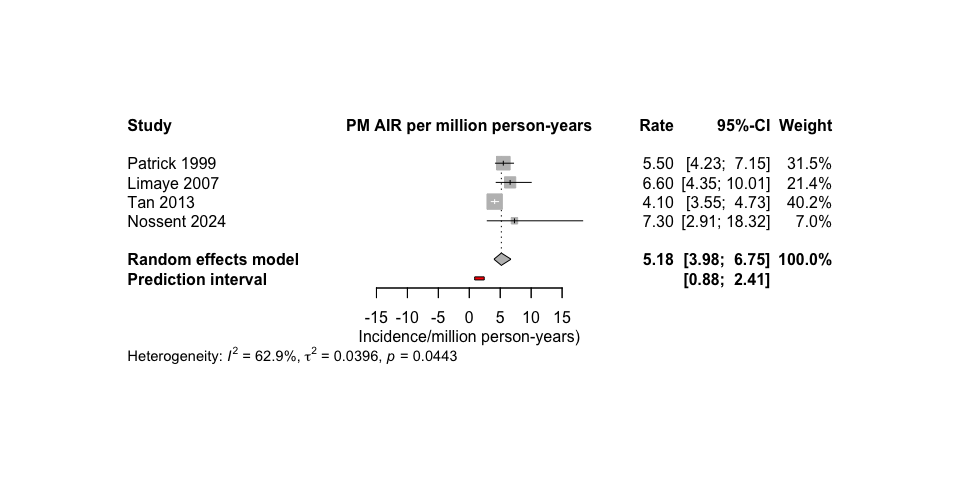


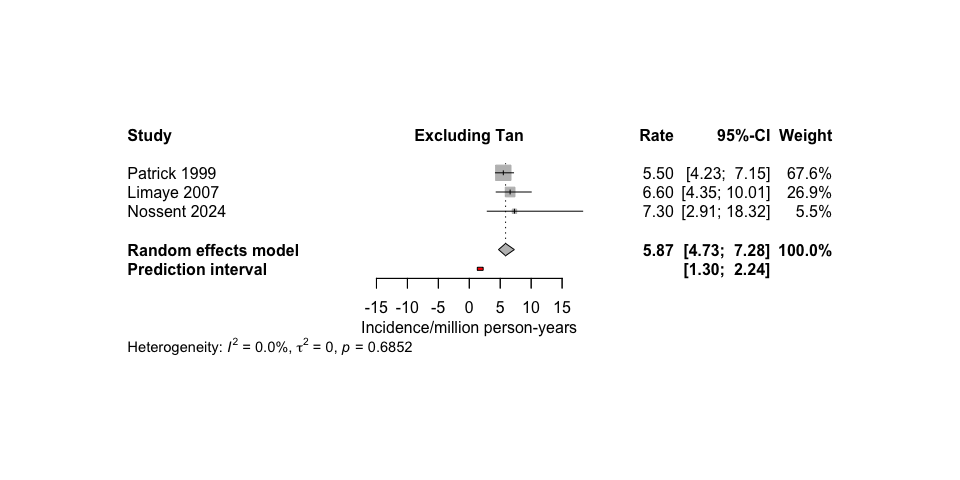


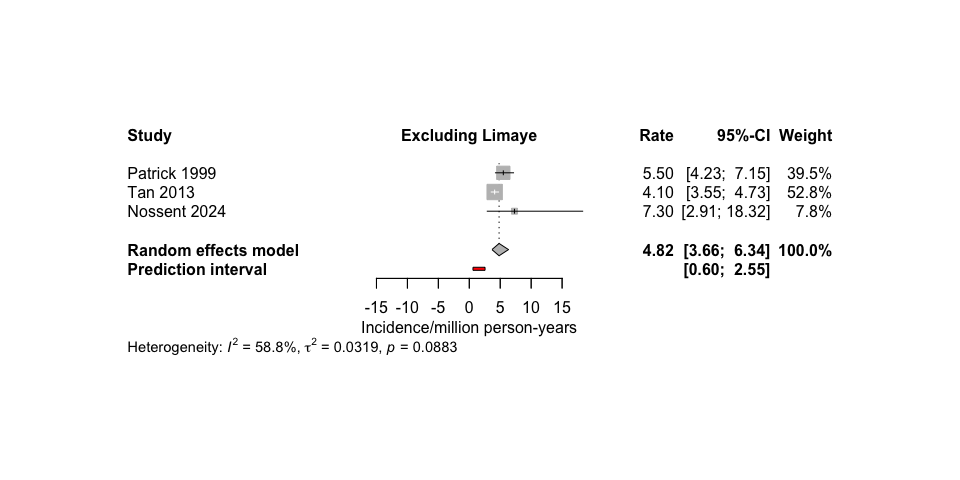
Excluding individual studies had only a modest effect on the pooled incidence rate (AIR) for polymyositis. The overall estimate was 5.18 (95% CI: 3.98–6.75) per million person-years. Removing Tan increased the estimate to 5.87, while removing Limaye decreased it to 4.82; these two studies were based on overlapping populations but used different case-finding methods. Excluding Nossent, assessed as moderate risk of bias, yielded a pooled AIR of 5.05. All estimates remained within the original confidence interval, suggesting the overall result is reasonably stable. Notably, the inclusion of two studies from a single geographic region did not materially alter the pooled estimate.

**6.6 Inclusion Body Myositis Prevalence**

The forest plot shows the pooled prevalence of IBM using the double arcsine transformation and a random-effects model, with back-transformation of pooled results for interpretability. Two studies (Patrick and Needham) relied on overlapping data sources for case identification, using records from the Australian Neuromuscular Research Institute and hospital biopsy databases in Western Australia. To explore the potential influence of this regional concentration on the pooled estimate, we conducted a sensitivity analysis. When both studies were included, the prevalence was 22.3 per million, excluding Phillips increased this to 27.7 and excluding Needham also increased this, to 25.1. This represented a change of < 2 per 100,000 but over 10%, therefore only the more recent study (Needham) was retained in the meta-analysis.
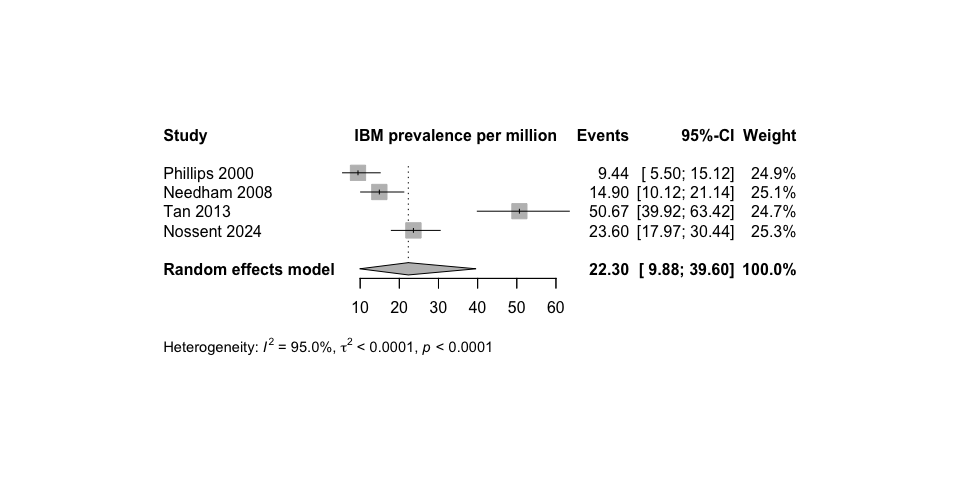


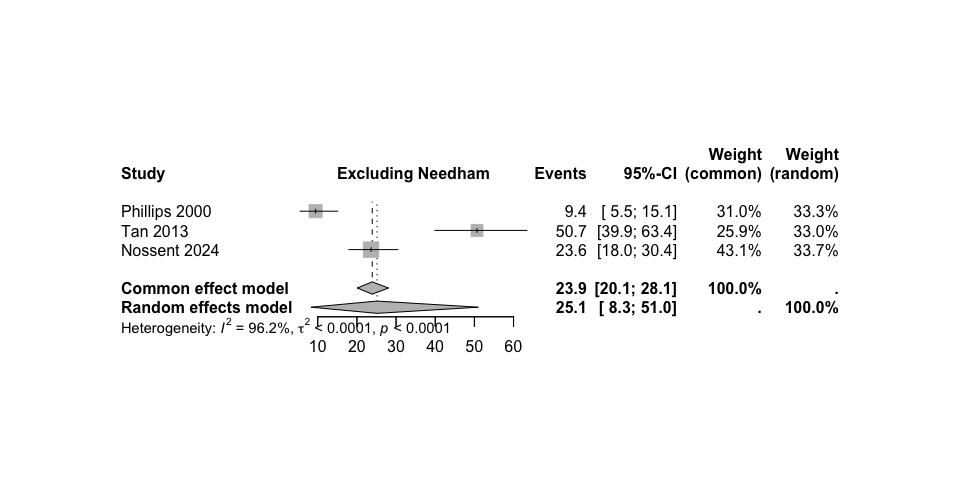

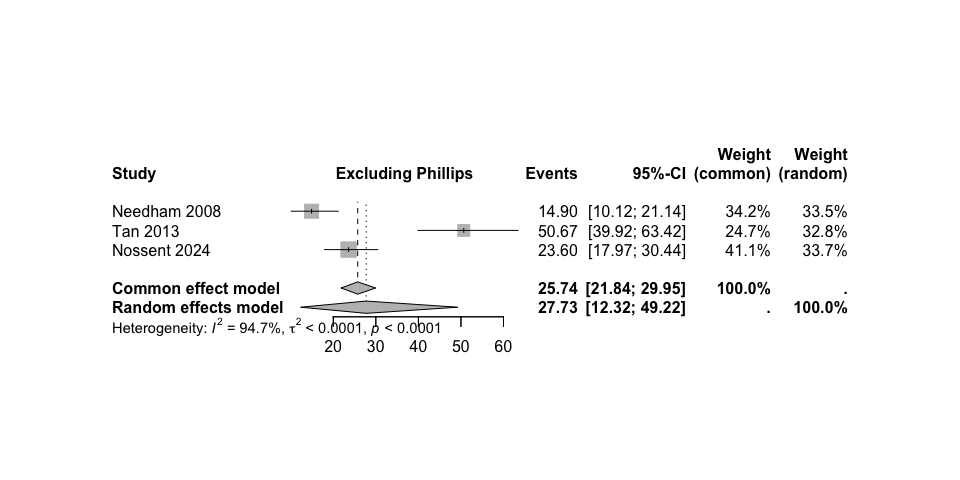


**7. SPONDYLOARTHRITIS**

**7.1 Spondyloarthritis Study Characteristics**

| **Study** | **Disease** | **Location** | **Period** | **Case identification** | **Disease definition** | **Data (I/P)** | **Risk of bias** |
| --- | --- | --- | --- | --- | --- | --- | --- |
| Nossent 2022 | Ankylosing Spondylitis | WA | 2020 | Identifed all WA patients prescribed a TNFi for AS on the PBS. Then assumed 60% of all AS popn took an TNFi to calculcate total WA prevalence | Meeting PBS criteria for TNFi for AS | P | Low-Mod |
| Minaur 2004 | Psoriatic Arthritis | QLD | 2002 | House to house survey through the community, until each person had either done the survey or refused to participate. | Clinician examination | P | High |
| Gonzalez-Chica 2018 | Spondyloarthropathy, unspecified | Aus | 2000 - 2016 | GP entered diagnosis of RA or spondyloarthropathy into their electronic medical records (MedicineInsight database). | GP diagnosis of spondyloarthritis, spondylosis, spondyloarthropathy | P | High |

**7.2 Spondyloarthritis Study Findings**

| **Study** | **Population denominator** | **Cases** | **Reported prevalence (%)** | **Included in Meta-Analysis** |
| --- | --- | --- | --- | --- |
| Nossent 2022 | All of WA: 2.5 million | 1925 | 0.29 | No |
| Minaur 2004 | All of Yarrabah Aboriginal Community: 847 | 4 | 0.5 | No |
| Gonzalez-Chica 2018 | Entire patient population of GPs enrolled in MedicineInsight databse: 1501267 | NR | 1.1 | No |

1. Nossent, J., et al., *Hospitalisation rates and characteristics for adult and childhood immunoglobulin A vasculitis in Western Australia.* Internal Medicine Journal, 2019. **49**(4): p. 475-481.

2. Nossent, J., et al., *The current role for clinical and renal histological findings as predictor for outcome in Australian patients with lupus nephritis.* Lupus, 2018. **27**(11): p. 1838-1846.

3. Nossent, J.C., et al., *Population-wide long-term study of incidence, renal failure, and mortality rates for lupus nephritis.* INTERNATIONAL JOURNAL OF RHEUMATIC DISEASES, 2024. **27**(2).

4. Ong, C., K. Nicholls, and G. Becker, *Ethnicity and lupus nephritis: an Australian single centre study.* Internal medicine journal, 2011. **41**(3): p. 270-278.

5. Statistics, A.B.o., *National Health Survey 2022*. 2023.

6. Koller-Smith, L., et al., *A novel method to monitor rheumatoid arthritis prevalence using hospital and medication databases.* 2023.

7. Almutairi, K., et al., *The prevalence of rheumatoid arthritis in Western Australia.* BMC rheumatology, 2022. **6**(1): p. 1-8.

8. González-Chica, D.A., et al., *Epidemiology of arthritis, chronic back pain, gout, osteoporosis, spondyloarthropathies and rheumatoid arthritis among 1.5 million patients in Australian general practice: NPS MedicineWise MedicineInsight dataset.* BMC musculoskeletal disorders, 2018. **19**: p. 1-10.

9. Statistics, A.B.o., *National Health Survey: First Results, 2014-15*. 2015.

10. Statistics, A.B.o., *National Health Survey: Summary of Results 2007-2008*. 2009.

11. Hill, C.L., et al., *Health related quality of life in a population sample with arthritis.* The Journal of rheumatology, 1999. **26**(9): p. 2029-2035.

12. Bellamy, N., et al., *Rheumatoid arthritis in twins: a study of aetiopathogenesis based on the Australian Twin Registry.* Annals of the rheumatic diseases, 1992. **51**(5): p. 588-593.

13. Nossent, J., et al., *The spectrum of idiopathic inflammatory myopathies in Western Australia: epidemiological characteristics and mortality over time.* Rheumatology International, 2024. **44**(2): p. 329-337.

14. Tan, J.A., et al., *Incidence and prevalence of idiopathic inflammatory myopathies in south australia: A 30-year epidemiologic study of biopsy-proven cases.* Arthritis and Rheumatism, 2011. **63**(10 SUPPL. 1).

15. Needham, M., et al., *Prevalence of sporadic inclusion body myositis and factors contributing to delayed diagnosis.* Journal of Clinical Neuroscience, 2008. **15**(12): p. 1350-1353.

16. Phillips, B.A., P.J. Zilko, and F.L. Mastaglia, *Prevalence of sporadic inclusion body myositis in Western Australia.* Muscle and Nerve, 2000. **23**(6): p. 970-972.

17. Limaye, V., et al., *The epidemiology of dermatomyositis in South Australia.* APLAR Journal of Rheumatology, 2007. **10**(2): p. 94-100.

18. Patrick, M., et al., *Incidence of inflammatory myopathies in Victoria, Australia, and evidence of spatial clustering.* JOURNAL OF RHEUMATOLOGY, 1999. **26**(5): p. 1094-1100.
